# Inhibition of tumor progression and M2 microglial polarization by extracellular vesicle-mediated microRNA-124 in a 3D microfluidic glioblastoma microenvironment

**DOI:** 10.7150/thno.60851

**Published:** 2021-09-27

**Authors:** Soohyun Hong, Jae Young You, Kyurim Paek, Jubin Park, Su Jin Kang, Eun Hee Han, Nakwon Choi, Seok Chung, Won Jong Rhee, Jeong Ah Kim

**Affiliations:** 1 Research Center for Bioconvergence Analysis, Korea Basic Science Institute, Chungbuk 28119, Republic of Korea.; 2 Center for Scientific Instrumentation, Korea Basic Science Institute, Chungbuk 28119, Republic of Korea.; 3 Program in Biomicro System Technology, Korea University, Seoul 02841, Republic of Korea.; 4 Department of Bioengineering and Nano-Bioengineering, Incheon National University, Incheon 22012, Republic of Korea.; 5 Brain Science Institute, Korea Institute of Science and Technology, Seoul 02792, Republic of Korea.; 6 KU-KIST Graduate School of Converging Science and Technology, Korea University, Seoul 02841, Republic of Korea.; 7 Division of Bio-Medical Science & Technology, University of Science and Technology, Daejeon 34113, Republic of Korea.; 8 School of Mechanical Engineering, Korea University, Seoul 02841, Republic of Korea.; 9 Division of Bioengineering, Incheon National University, Incheon 22012, Republic of Korea.; 10 Department of Bio-Analytical Science, University of Science and Technology, Daejeon 34113, Republic of Korea.

**Keywords:** glioblastoma, microglia, extracellular vesicle, microRNA-124, three-dimensional (3D) cell culture

## Abstract

**Background:** Glioblastoma (GBM) is one of the most aggressive types of brain cancer. GBM progression is closely associated with microglia activation; therefore, understanding the regulation of the crosstalk between human GBM and microglia may help develop effective therapeutic strategies. Elucidation of efficient delivery of microRNA (miRNA) via extracellular vesicles (EVs) and their intracellular communications is required for therapeutic applications in GBM treatment.

**Methods**: We used human GBM cells (U373MG) and human microglia. MiRNA-124 was loaded into HEK293T-derived EVs (miR-124 EVs). Various anti-tumor effects (proliferation, metastasis, chemosensitivity, M1/M2 microglial polarization, and cytokine profile) were investigated in U373MG and microglia. Anti-tumor effect of miR-124 EVs was also investigated in five different patient-derived GBM cell lines (SNU-201, SNU-466, SNU-489, SNU-626, and SNU-1105). A three-dimensional (3D) microfluidic device was used to investigate the interactive microenvironment of the tumor and microglia.

**Results**: MiR-124 EVs showed highly efficient anti-tumor effects both in GBM cells and microglia. The mRNA expression levels of tumor progression and M2 microglial polarization markers were decreased in response to miR-124 EVs. The events were closely related to signal transducer and activator of transcription (STAT) 3 signaling in both GBM and microglia. In 3D microfluidic experiments, both U373MG and microglia migrated to a lesser extent and showed less-elongated morphology in the presence of miR-124 EVs compared to the control. Analyses of changes in cytokine levels in the microfluidic GBM-microglia environment showed that the treatment with miR-124 EVs led to tumor suppression and anti-cancer immunity, thereby recruiting natural killer (NK) cells into the tumor.

**Conclusions:** In this study, we demonstrated that EV-mediated miR-124 delivery exerted synergistic anti-tumor effects by suppressing the growth of human GBM cells and inhibiting M2 microglial polarization. These findings provide new insights toward a better understanding of the GBM microenvironment and provide substantial evidence for the development of potential therapeutic strategies using miRNA-loaded EVs.

## Introduction

Glioblastoma multiforme (GBM) is one of the most common and aggressive types of brain cancer. GBM is well known for its poor prognosis and high mortality with a markedly low 5-year relative survival rate 1-3. GBM constitutes a highly complicated and dynamic *in vivo* tumor-microenvironment (TME) that modulates and participates in GBM proliferation, invasion, and resistance to drugs. For a better proliferative expansion, GBM recruits and utilizes surrounding stromal cells and resident glial cells, such as astrocytes, oligodendrocytes, and microglia, by releasing various cytokines and chemokines 4, 5. Among glial cells, microglia may play a critical role in GBM 2, 6-8. Microglia are macrophages residing in the central nervous system (CNS), and they maintain resting status under homeostatic conditions until they are activated into either the M1 or M2 phenotype in response to different stimuli. Microglia have been classically subdivided into M1 and M2 phenotypes to characterize them as having either anti-tumor or pro-tumor properties. Recent studies have found that tumor-related microglia, also known as tumor-associated microglia (TAM)/glioma-associated microglia (GAM), acquire M2-like characteristics. Thus, regulation of the crosstalk between GBM and microglia can be an important strategy for cancer treatment 2, 8, 9.

Accumulating evidence shows that miRNAs play essential roles in complex TMEs, including GBM 10-12. MicroRNAs (miRNAs), small-noncoding RNA molecules, play a pivotal role in the progression of various cancers and have been proposed as novel targets for anti-cancer therapy. According to previous studies, one miRNA can bind to multiple targets and simultaneously suppress the translation of multiple genes to control biological processes, thereby leading to significant therapeutic outcomes in heterogenic cancers 12-16. Among these miRNAs, miR-124 is enriched in the normal brain, but its levels are downregulated in GBM 17-19. However, few studies have focused on the mechanism by which miR-124 regulates the crosstalk between GBM and immune cells. The role and mechanism of miR-124 in regulating tumorigenesis and immune response should be further elucidated 20, 21.

Despite the high potential of miRNAs, there are many challenges to their use as cancer therapeutics. Due to the sensitive and unstable properties of miRNAs, such as hydrophilic properties and negative charge, direct cell membrane penetration is difficult. This challenge can be solved by drug carriers such as extracellular vesicles (EVs) 22. Besides the biocompatibility, low toxicity, stability, and high efficacy, EVs have attracted significant attention due to their potential use in the delivery of exogenous drugs for brain-related diseases because they can cross the blood-brain barrier (BBB) 23-25. In this respect, researchers are interested in EVs as a potential miRNA delivery system for treating GBM 26.

Based on the current literature, the EV-mediated delivery of miRNAs is very promising. However, for precise assessment of the therapeutic effects of miRNAs delivered via EVs, a reliable *in vitro* system is required to test the efficacy of EV drugs in preclinical evaluations. As an effective alternative, 3D microfluidic models have been suggested to recapitulate the structure, function, and physiological features of specific tissues and to mimic the high complexity and spatial heterogeneity of the *in vivo* microenvironment. The 3D microstructure in a device employs a gel-supported extracellular matrix, which allows the observation of dynamic and phenotypic changes of cells in response to various chemical factors induced by cell-cell and cell-extracellular matrix interactions. Thus, the 3D microfluidic system can be used as an alternative translational tool to bridge the gap between preclinical models and clinical outcomes, as well as to present a well-suited model for testing the EV drugs 27-29.

In this study, we addressed several questions on whether miR-124-loaded EVs (hereafter miR-124 EVs) could be effective anti-cancer drugs for the treatment of GBM. We investigated potential functional targets of miR-124 involved in tumor growth, as well as microglia polarity. Further, we investigated the mechanism by which miR-124 delivered via EVs regulated the related pathways in both the tumor and microglia. We explored the role of miR-124 EVs in GBM by simulating their therapeutic effects using a 3D microfluidic device as a translational tool to verify the anti-cancer and anti-M2 microglial polarization effects of miR-124 EVs. Changes in tumor-microglia interactions also affected the NK cell behavior. Our findings may provide new insights into a better understanding of GBM-immunity interaction and for the development of a novel therapeutic strategy for GBM.

## Results and discussion

### Overall research strategy

GBM is one of the most aggressive and invasive forms of brain cancer, which is highly resistant to anti-cancer drugs. To increase therapeutic outcomes and develop anti-cancer drugs, a detailed understanding of the GBM and its surrounding microenvironment, including the immune system, is required 2, 30. Despite the considerable interest in tumors and the immune microenvironment, there are few reports on the role of microglia. Recent studies have focused on the potential role of miRNAs in mediating the crosstalk between microglia and GBM, and emerging evidence suggests that miRNAs have various essential roles in brain-related diseases 10, 15. Among others, reports indicate that miR-124 plays a key role in the regulation of various cells in the brain 8, 20, 21. Reportedly, normal brain tissue has a significantly higher expression of miR-124 compared to that in GBM tissues. Furthermore, miR-124 reportedly suppresses the tumor and stabilizes microglial cells 17, 30-32. These findings suggest that the dysregulation of miR-124 may disturb the homeostasis in the healthy brain, thereby accelerating glioma pathogenesis.

**Figure 1:**
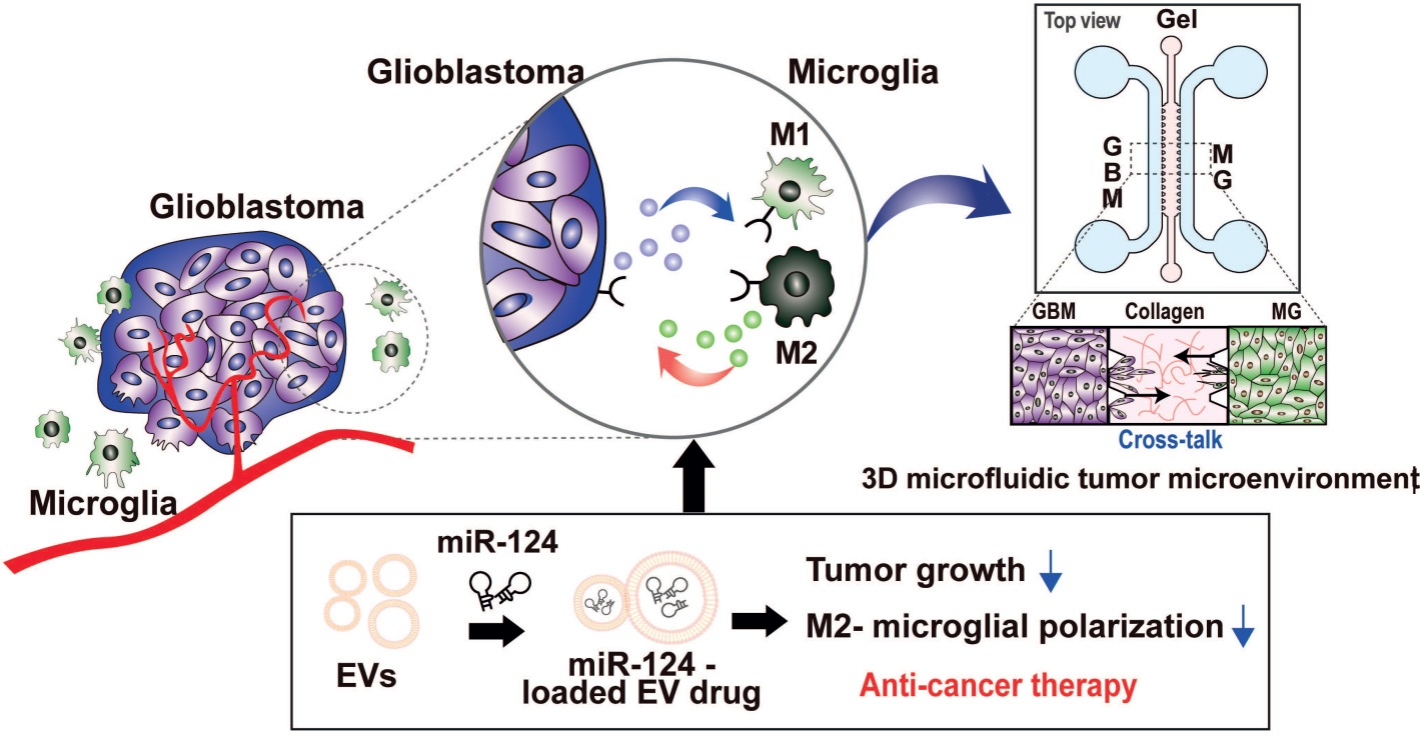
Schematic illustration showing the overall experimental strategy of miR-124-loaded extracellular vesicle (EV) delivery into glioblastoma (GBM) and microglia (MG) for anti-cancer therapy. The proliferation of GBM and M2 polarization of microglia were inhibited by miR-124 delivered via EVs. Drug efficacy of miR-124 delivered via EVs in the interactive microenvironment of GBM-microglia was evaluated through an *in vitro* 3D microfluidic device.

Thus, our strategy was to deliver miR-124 to the GBM microenvironment via EVs as an efficient miRNA delivery vehicle, as shown in the scheme in Figure 1. We expected the delivered miR-124 to downregulate both the downstream signaling pathway related to tumor progression and M2 microglial polarization, a tumor-supportive phenotype, ultimately inhibiting tumor growth and tumor-favorable microglial status in the GBM microenvironment. To precisely investigate the phenomena that occur in the GBM microenvironment, we recreated the local GBM microenvironment by co-culturing GBM cells and microglia in a 3D microfluidic device, and we studied the mechanism by which miR-124 delivered by EVs influenced tumor progression while interacting with microglia. Moreover, we investigated targets and pathways that may be involved in the regulation of tumorigenesis and microglial polarization. In the following sections, we describe how we validated that tumor proliferation and M2 microglial polarization can be modulated through EV-based miR-124 delivery in the GBM microenvironment, thereby inducing synergistic anti-cancer therapeutic effects.

### EV characterization

HEK293T-derived EVs were used as carriers to load miRNAs. Recent studies showed that few molecules involved in the disease- or cancer-related pathways are enriched in HEK293T EVs 33. Also, it was demonstrated that HEK293T cell-derived EVs are overall benign in terms of immune response and toxicity in animal studies 34. Thus, the inadvertent delivery of unwanted molecules is minimized, thereby reducing the potential *in vivo* side effects. We have previously reported the optimization of EV isolation, purification, and delivery of exogenous miRNAs into carrier EVs, as well as characterization of the loading efficiency, and miRNA stability 27. Furthermore, we previously demonstrated that a high amount of EVs was uptaken by cells, and miRNAs loaded in the EVs can efficiently be delivered to cells 27, 35, 36. Under the same conditions, EVs were isolated from the HEK293T-conditioned media using the ExoQuick method (EXQ), loaded with miRNAs, and purified via ultrafiltration (UF), for successful preparation of miRNA EVs. We evaluated the extent to which the EVs protected the miRNAs from degradation. The miRNA-lipo which carried the same amount of miRNAs loaded using lipofectamine, and miRNA EVs were incubated at 37 °C for 48 h, and then, the miRNA level was quantified (Figure S1). As a result, the expression level of EV-enclosed miRNAs was very stable (80% of the original level was maintained on average). This indicated that EVs are suitable vehicles for the efficient and stable delivery of miRNAs.

We also identified the concentration and size distribution, as well as the zeta potential of EVs through nanoparticle tracking analysis (NTA) after miRNA loading (Figure S2A-S2F). The sizes of HEK293T-derived carrier EVs ranged from 50-150 nm and the surface charges of the miRNA EVs were similar to those of original EVs. The average size of EVs without miRNAs was 86 nm (Figure S2A), and the average size of miRNA EVs was 77-87 nm (Figure S2B-S2D). This indicated that miRNA loading did not significantly alter the physical properties of the EVs. However, compared to HEK293T-derived EVs, the size of GBM (U373MG cell)-derived EVs were much bigger (average 190 nm), which was slightly larger than the typical size range of EVs (Figure S2E). There were no significant differences in the zeta potential of the EVs before and after miRNA loading (Figure S2F). Further, transmission electron microscopy (TEM) analysis of the EVs showed a round morphology (Figure S2G-S2H), which indicated that the EVs remained intact after miRNA loading. However, the sizes of the EVs determined using TEM were relatively smaller than those determined using NTA analysis, possibly due to shrinkage during TEM sample preparation 34. Additionally, western blot analysis showed that the EV markers, such as CD63 and CD81 were detected in the carrier EVs and miRNA EVs (Figure S2I). No apparent differences in the amounts of EV surface proteins were observed. CD63 and CD81 are highly expressed tetraspanins on the surface of EVs and are considered as surface protein markers for EVs 37, 38. Because no change was observed in the amount of these membrane proteins during miRNA loading, it was predicted that the EV membrane was still intact, and thus the EV integrity was maintained even after miRNA loading. Thus, EVs produced based on the methods we described herein were used as therapeutic tools in subsequent experiments.

### Anti-proliferation effect of miRNA EVs on GBM

Before selecting miR-124, we screened miRNA candidates from the literature. We sorted miR-124, miR-128, miR-let-7c, and miR-34a, which reportedly regulate pathological pathways in GBM or microglia as per several studies 10, 15, 17, 19, 20, 39-44. However, few of the reported miRNAs have not been implicated in the regulation of the crosstalk between GBM and microglia.

First, to verify the anti-proliferative effect of the selected miRNAs in GBM, we investigated the anti-proliferative effects of four different miRNAs on human GBM cell lines U373MG and U87MG using the CCK-8 assay. For the preliminary screening of candidates, cells seeded in 96-well plates were transfected with miRNAs using lipofectamine (hereafter lipofection, miRNA Lipo). After 72 h, the comprehensive results showed that 20 nM of miR-124 and 20 nM of miR-128 decreased the proliferation rate to 80-85% in both U373MG and U87MG cell lines (*p* < 0.05) (Figure S3A-S3B). In the case of miR-34a, there was approximately an 8 and 12% reduction of proliferation in both U373MG and U87MG, respectively, but the change was not significant (p > 0.05). Additionally, miR-let-7c did not show an anti-proliferative effect in this study. Based on this, miR-124 and miR-128 showed the most positive anti-proliferative effects in U373MG and U87MG.

**Figure 2:**
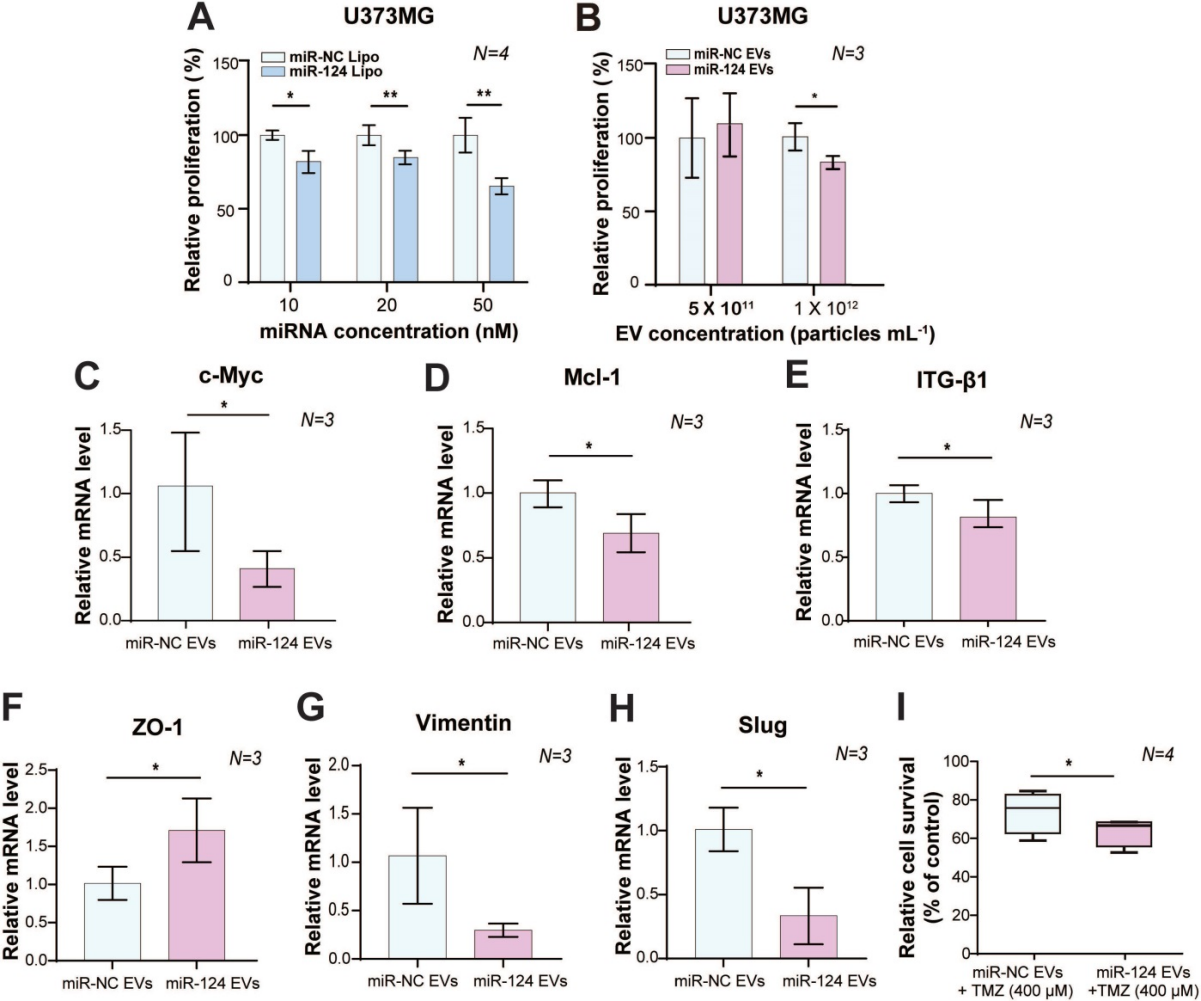
** Anti-tumor effect of miR-124 in GBM cells. (A)** The anti-proliferative effect of miR-124 introduced using lipofection on U373MG cells. Proliferation assay was performed 72 h after U373MG cells had been transfected with miRNA at 10, 20, and 50 nM. **(B)** The anti-proliferative effect of miR-124 EVs in U373MG cells. Two different concentrations of EVs (5×10^11^ particles mL^-1^ and 1×10^12^ particles mL^-1^) were added to U373MG cells for 72 h. The miR-NC EVs were used as a control. **(C-I)** Relative mRNA expression level in U373MG was affected by miR-124 EVs (1×10^12^ particles mL^-1^). **(C and D)** Proliferation markers, *c-Myc* and *Mcl-1*; **(E)** migration-associated marker, *ITG-β1*; **(F)** epithelial marker *ZO-1*; **(G and H)** mesenchymal markers, *Vimentin* and *Slug*. **(I)** Changes in the chemosensitivity of U373MG cells upon miR-124 EV treatment in the presence of temozolomide (TMZ, 400 μM). Cells were treated for 48 h. GAPDH was used as an internal control. All values are expressed as the mean ± S.D. (* *p* < 0.05, ** *p* < 0.01).

Next, we examined the dose-dependent anti-proliferative effects of miR-124 and miR-128 on GBM cells. We found that miR-124 Lipo showed a significant anti-proliferative effect in a dose-dependent manner in both U373MG **(**Figure 2A) and U87MG cells (Figure S4A-S4B), with better sensitivity in U373MG cells. At concentrations higher than 20 nM, anti-proliferative effects of miR-124 Lipo were evident (Figure 2A, *p* < 0.05, *p* < 0.01, or *p* < 0.001). By contrast, although miR-128 Lipo showed anti-proliferative effects at certain concentrations, it did not show a dose-dependent anti-proliferative effect in U87MG cells (Figure S5A-S5B).

With EV delivery, miR-124 EVs showed a significant anti-proliferative effect compared to miR-negative control (miR-NC) EVs at a concentration of 1×10^12^ particles mL^-1^ (*p* < 0.05) (Figure 2B). However, there was no anti-proliferative effect on U87MG cells at this concentration (Figure S4B, *p* > 0.05). The miR-128 data were not consistent as responses were not dependent on the concentration of miR-128 EVs in both U373MG and U87MG cell lines. Instead, U87MG treated with miR-128 EVs showed 33% more proliferation than the control group (Figure S5C). Taken together, we concluded that the miR-124 Lipo and miR-124 EVs had consistent and positive anti-proliferative effects on GBM cell lines, with better efficacy in U373MG cells. We confirmed that miR-124 EVs suppressed proliferation in GBM, and subsequently optimized experimental conditions to 1×10^12^ particles mL^-1^ of EVs in U373MG. Although treatment with a higher EV concentration than 1×10^12^ particles mL^-1^ is expected to improve anti-tumorigenesis, it was not evaluated in this study due to the limited EV productivity by the cells and the lack of EV concentration methods that ensure high EV yield. Also, we observed that the delivery of a high concentration of miRNA might have a rather negative effect compared to lower concentrations in certain types of cell lines (Figure S5C). Future studies are, therefore, needed to clarify the differential anti-proliferation effects of miRNAs using a different delivery method, such as lipofection and EV delivery.

### Anti-tumor effect of miR-124 EVs on GBM

Besides the fast growth rate, GBM is also known to invade and diffuse swiftly into surrounding healthy regions of the brain, which results in poor prognosis and low survival rates 1, 7, 30. Based on the optimized concentration of EVs shown above, we investigated the therapeutic effects of miR-124 EVs by assessing the mRNA levels of genes related to proliferation, migration, and epithelial-mesenchymal transition (EMT) in GBM. The mRNA expression levels of proliferation markers, the proto-oncogene *c-Myc*, and the anti-apoptotic marker *Mcl-1* were significantly downregulated (*p* < 0.05). (Figure 2C-2D). Expression of migration-related marker integrin beta-1 (*ITG-β1*) showed a 0.75-fold reduction (*p* < 0.05) upon miR-124 EV treatment (Figure 2E). There are several mechanisms involved in tumor cell motility, such as mesenchymal, amoeboid, and collective movements. Tumor cells utilize diverse strategies during migration, and EMT is one of the well-studied migration modes implicated in the phenotypical and functional plasticity of tumor progression. Besides, GBM expansively proliferates mainly through mesenchymal movements 44, 45. EMT markers in tumors, thus, represent migration and invasion tendency, expression levels of which are used to estimate metastasis. Upon miR-124 EV treatment, expression levels of the epithelial marker *ZO-1*, and mesenchymal markers *Vimentin* and *Slug*, showed a 1.7-fold increase, a 0.3-fold decrease, and a 0.33-fold decrease, respectively (*p* < 0.05) (Figure 2F-2H). These results implied that EMT in U373MG was reduced via the EV delivery of miR-124; therefore, EMT-associated processes in GBM, including metastasis, were expected to decrease. Further, we compared the survival rate of U373MG cells treated with miR-124 + temozolomide (TMZ) with that of cells treated with miR-NC + TMZ. U373MG cells were pre-tested with TMZ in a dose-dependent manner (25-400 μM) (Figure S6). As a result, the survival rate of U373MG cells was significantly reduced with the treatment of miR-124 EVs + TMZ (400 μM) (Figure 2I). These results suggest that miR-124 EV enhances the chemosensitivity of U373MG to TMZ and improves the anti-proliferation effect of the drug.

### M2 polarization-suppressing effect of miR-124 EVs on human microglial cells

In the human GBM microenvironment, GBM cells release various chemoattractants to recruit immune cells. The immune cells also release numerous cytokines/chemokines depending on the microenvironment. This complex interaction creates a pro- or an anti-GBM microenvironment 8, 30. Microglia reside in the CNS and exhibit a plastic phenotype according to the microenvironmental conditions. Upon stimulation by cytokines in the TME, microglia change their phenotypes to a pro-inflammatory M1 or an anti-inflammatory M2 phenotype 46, 47. TAM/GAM accounts for a third of the tumor mass, and most exhibit M2-like polarized microglial characteristics, including the release of anti-inflammatory cytokines, angiogenic factors, and proteases. Once microglia become TAM/GAM through stimulation by chemoattractants from GBM, M2-polarized microglia release cytokines, such as TGF-β or IL-10, which contribute to creating a tumor growth supportive environment 5.

In this study, we investigated whether miR-124 EVs regulate the polarization of microglia in the GBM microenvironment. We hypothesized that GBM-derived EVs (hereafter GBM EVs) induce immune cell reprogramming in favor of cancer growth and metastasis by activating the microglia through the cargo carried by these EVs 48, 49. We found that GBM EVs tended to induce M2 microglia rather than M1 when microglia were treated with GBM EVs isolated from the U373MG-conditioned media (Figure S7).

**Figure 3:**
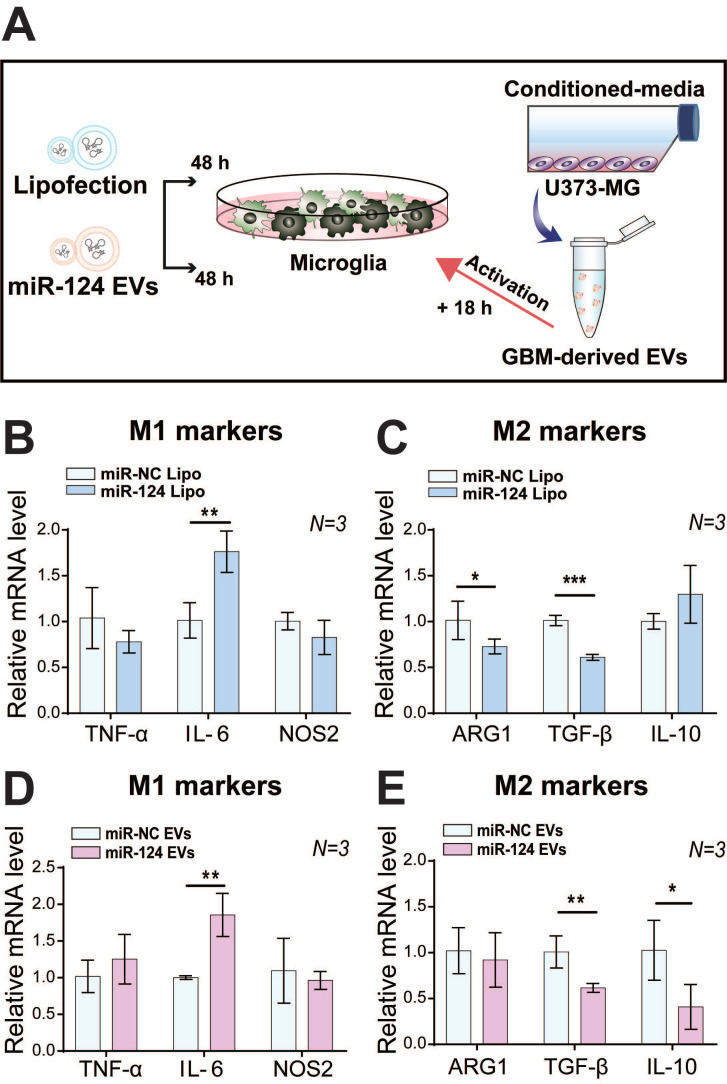
** Inhibition of M2 polarization in microglia by miR-124. (A)** Microglia were transfected with miR-124 using (**B and C**) lipofection or (**D and E**) EV delivery. The relative mRNA expression M1 (*TNF-α*, *IL-6*, and *NOS2*) and M2 polarization-associated markers (*ARG1*, *TGF-β*, and *IL-10*) was investigated. Microglia were treated with miR-124 (20 nM) or miR-124 EVs (1×10^12^ particles mL^-1^) for 48 h and further stimulated with GBM EVs (3×10^9^ particles mL^-1^) for 18 h. GAPDH was used as an internal control. All values are expressed as the mean ± S.D. (* *p* < 0.05, ** *p* < 0.01, *** *p* < 0.001).

We investigated the effect of miR-124 on microglial polarization with miR-124 lipofection or miR-124 EVs for 48 h, which were further stimulated with GBM EVs for 18 h (Figure 3A). Interestingly, the expression level of the M1 marker *IL-6* was significantly upregulated (1.76-fold, *p* < 0.01), whereas the expression level of M2 markers *TGF-β* and *ARG1* were significantly downregulated (0.73 and 0.61 folds, respectively) upon miR-124 lipofection. The expression of other markers was not significantly different (*p* > 0.05) (Figure 3B-3C). Treatment with miR-124 EVs showed a similar pattern, which significantly upregulated *IL-6* (1.86-fold, *p* < 0.01), and downregulated *TGF-β* and *IL-10* (0.62 and 0.41 folds, respectively) (Figure 3D-3E). Overall, we revealed that miR-124 EVs suppressed M2 microglial polarization.

### STAT3-regulating effect of miR-124 EVs in GBM and microglial cells

**Figure 4:**
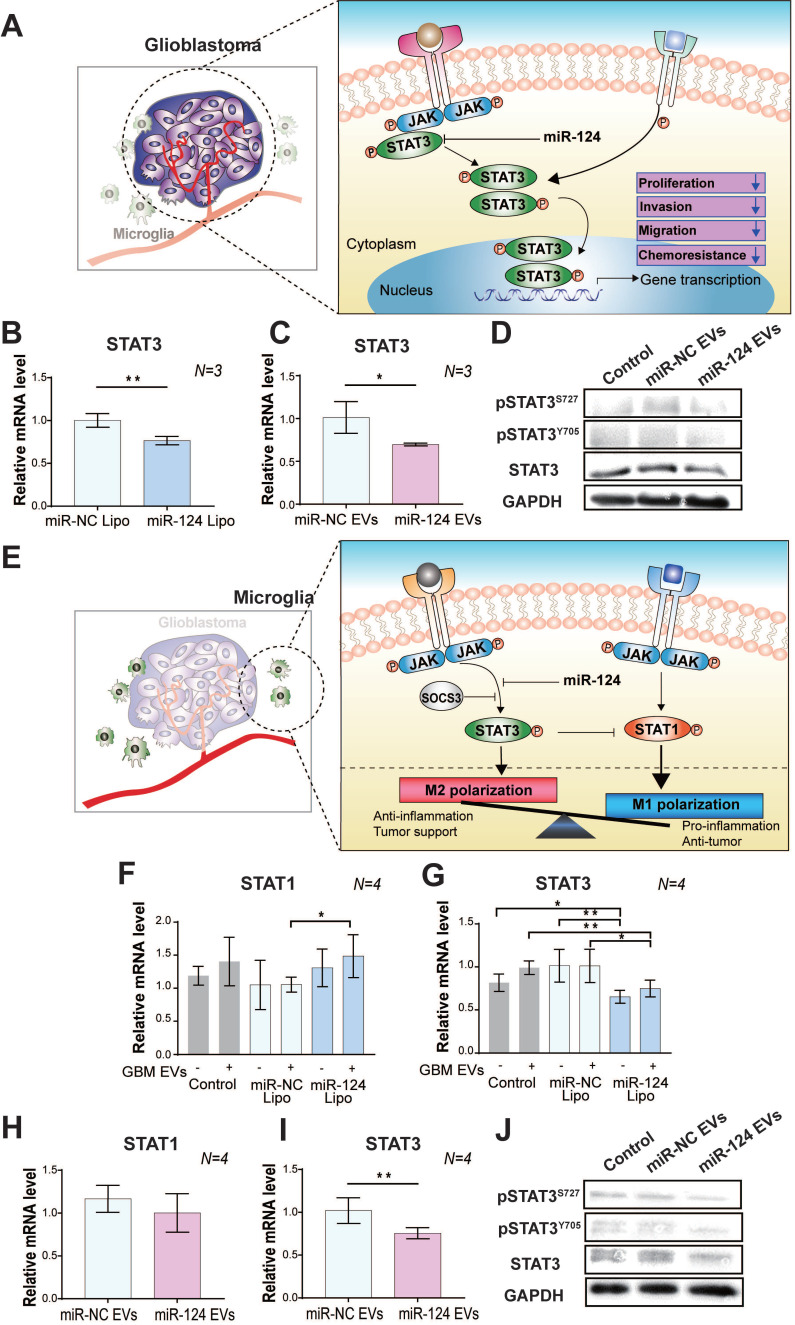
** The regulatory effect of miR-124 EVs on STAT3 in (A-D) GBM and (E-J) microglia. (A)** A schematic illustration showing the regulation of the STAT3-mediated tumor progression by miR-124 in GBM. MiR-124 downregulates STAT3 expression. The relative mRNA levels of *STAT3* was determined in U373MG cells after (**B**) lipofection with 20 nM miR-124 or (**C**) treatment with miR-124 EVs (1×10^12^ particles mL^-1^). U373MG was lipofected with miRNAs for 72 h, or treated with miR-124 EVs for 48 h. **(D)** Western blot analysis of STAT3 activation by phosphorylation at Tyr705 (pSTAT^Y705^) and Ser727 (pSTAT^S727^) in U373MG cells. GAPDH was used as an internal control. **(E)** A schematic illustration showing regulation of the STAT3-mediated microglial M1 and M2 polarization by miR-124. MiR-124 downregulates STAT3 expression and may trigger STAT1 expression. The balanced and controlled expression/activation of STAT1 and STAT3 is a key regulator for determining of the M1/M2 phenotype. Changes in the relative expression levels of *STAT1* and *STAT3* in microglia by miR-124. These expression levels were determined after (**F and G**) lipofection with 20 nM miR-124 or (**H and I**) treatment with miR-124 EVs (1×10^12^ particles mL^-1^). Microglia were treated with miRNAs for 48 h and further stimulated with GBM EVs (3×10^9^ particles mL^-1^) for 18 h. **(J)** Western blot analysis of STAT3 activation by phosphorylation at Tyr705 (pSTAT^Y705^) and Ser727 (pSTAT^S727^) in microglia. GAPDH was used as an internal control. All values are expressed as the mean ± S.D. (* *p* < 0.05, ** p < 0.01).

Upon observation that miR-124 EVs had anti-tumor and anti-M2 microglial polarization effects in the GBM microenvironment, we next investigated the pathway involved in these events. Accumulating evidence 50, 51 indicates that STAT3 is highly involved in various major hallmarks of tumor aggressiveness, and plays an essential role in regulating cell proliferation, growth, migration, and invasion (Figure 4A). Primarily, persistent activation of STAT3 is associated with EMT and a bad prognosis in cancer 51, 52. STAT3 is also involved in the regulation of its downstream genes, such as *Mcl-1*, *VEGF*, and *c-Myc*. Therefore, regulation of the STAT3 expression can be a promising anti-cancer therapy. Several recent studies have also reported that miR-124 targets *STAT3*, which negatively controls its expression 53, 54. We performed quantitative real-time RT-PCR (qRT-PCR) to evaluate the mRNA expression level of *STAT3* in GBM. The results showed that miR-124 Lipo (20 nM for 72 h) negatively regulated *STAT3* expression in U373MG. The mRNA expression levels of *STAT3* decreased by 0.77 fold (*p* < 0.05) in the miR-124-treated group compared to that in the miR-NC group (Figure 4B). This effect was also observed upon the miR-124 EV treatment (1×10^12^ particles mL^-1^ for 48 h). *STAT3* mRNA expression level decreased by 0.7 folds (*p* < 0.05) in the miR-124 EV group compared to that in the miR-NC EV group (Figure 4C). The binding of the activating factors to their receptors on the cell surface induces receptor dimerization and activation of the tyrosine kinase Janus-activated kinase (JAK), followed by subsequent phosphorylation of STAT3 at Tyr705 or Ser727. Phosphorylated STAT3 dimerizes and migrates to the nucleus, and activates transcription of genes that modulates various cellular processes, such as proliferation, migration, and inflammatory responses. Owing to the pro-tumorigenic activities of STAT3, its activation is involved in multiple human malignancies. In the present study, miR-124 EVs not only directly reduced the expression of total STAT3 but also inhibited the phosphorylation of STAT3 at both Tyr705 (Y705) and Ser727 (S727) (Figure 4D). It is well known that the phosphorylation site of STAT3 determines the signal transduction cascade in cancer progression, but the dominant phosphorylation sites in this pathway have not been fully elucidated. Based on these results, we confirmed that miR-124 significantly downregulated STAT3 so that tumor progression activity could be successfully reduced. This provides strong evidence that miR-124 EVs modulates STAT3 activity in GBM cells and may have therapeutic effects.

In microglia, STAT3 is also an essential factor that induces M2 activation 52. The expression of STAT3 affects the expression level of other transcription factors like STAT1, which is suggested to be a tumor suppressor. M1/M2 polarization depends on the balance in STAT1/STAT3 expression 55. Thus, targeting STAT3 using miR-124 may be a key therapeutic approach to suppress the proliferation of GBM and to prevent M2 polarization of microglia, thereby disturbing the dynamic crosstalk between GBM and microglia. (Figure 4E). Thus, we examined the mRNA expression level of *STAT1/STAT3* in miR-124 Lipo or miR-124 EV-treated microglia. In microglia with miR-124 Lipo, the mRNA expression of *STAT1* was upregulated, whereas that of *STAT3* was downregulated. (Figure 4F-4G). In particular, *STAT3* downregulation was remarkable in microglia even without GBM EVs (Figure 4G), thereby indicating that miR-124 might inhibit STAT3 even in the resting state. In the case of the miR-124 EV treatment, the change in *STAT1* expression was not significant (*p* > 0.05) (Figure 4H), but *STAT3* was significantly downregulated by 0.75 folds (*p* < 0.05) (Figure 4I). Similarly, both total STAT3 and phosphorylated STAT3 expression in microglia were inhibited at the protein level upon miR-124 EV treatment (Figure 4J). Therefore, it is evident that miR-124 delivered by EVs into microglia effectively targets STAT3 and negatively regulates STAT3 signaling by suppressing mRNA expression and translation.

Based on these results, miR-124 was delivered to cells successfully via EVs, which resulted in the significant inhibition of STAT3 expression, which is a potent key regulator in GBM-microglia. Although miR-124 is a clear negative regulator of STAT3 signaling, the results on its role in STAT1 regulation are not conclusive. In our experiments, *STAT1* expression was not changed in GBM regardless of the delivery method **(**Figure S8A-S8B**)**. However, in microglia, STAT1/STAT3 balance was affected by miR-124, which might have induced a certain type of polarization. Downregulation of STAT3 may trigger STAT1 upregulation. Thus, future studies should focus on delineating the mechanism underlying the regulation and proper STAT1/STAT3 balancing by miRNAs. Nevertheless, it is evident that miR-124 EV has synergistic anti-cancer effects on both GBM and microglia via suppression of STAT3 expression, which provides highly promising therapeutic opportunities for treating GBM.

### GBM and microglia co-culture in a 3D microfluidic system

**Figure 5:**
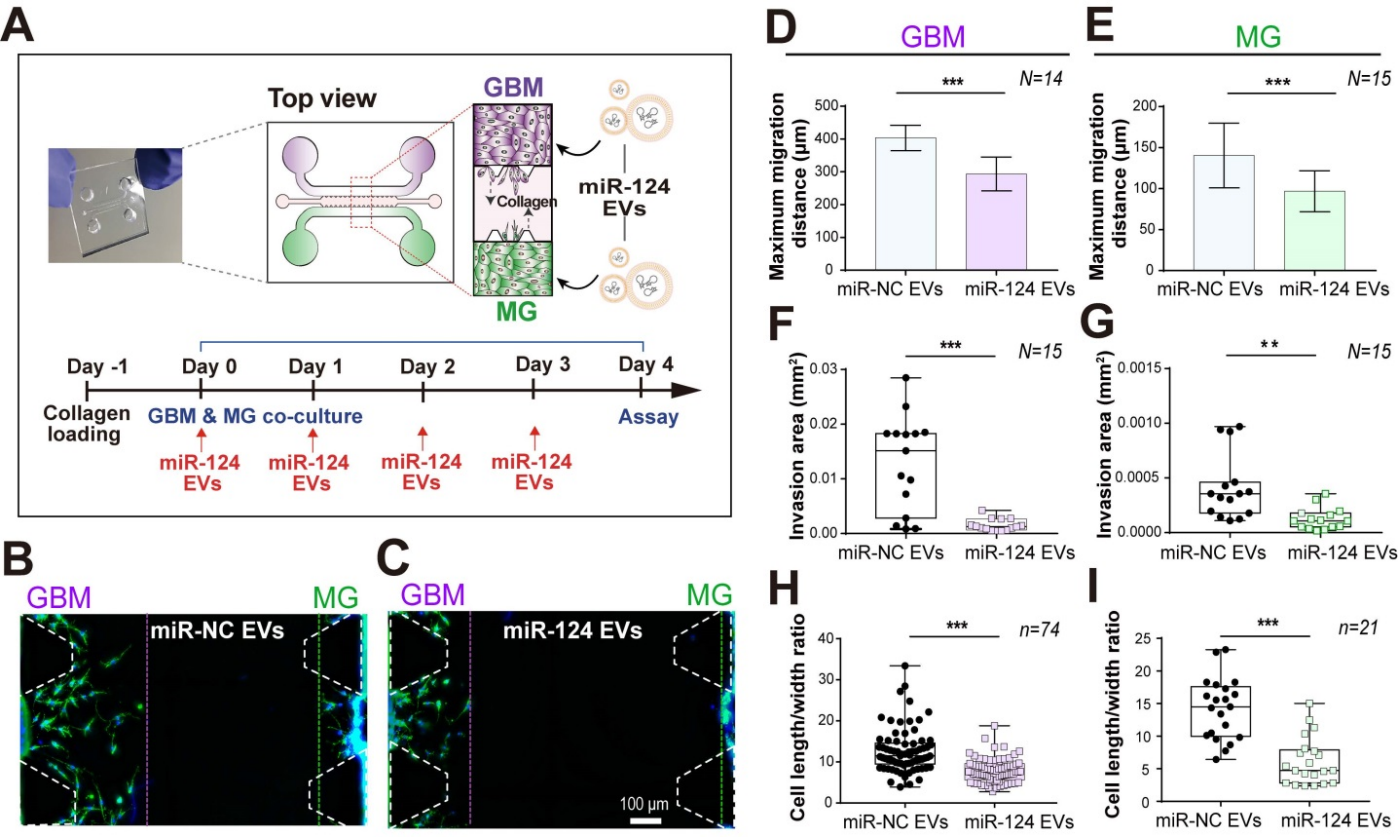
** A 3D microfluidic co-culture device to recreate the interaction between GBM and microglia. (A)** U373MG and microglia were co-cultured for 4 days with miRNA EVs. **(B and C)** The representative images (on day 4) presented a phenotypic change in U373MG co-cultured cells with microglia in a microfluidic device with a culture medium containing (B) miR-NC EVs or (C) miR-124 EVs. Cells were immunostained for F-actin (Phalloidin, green) and nuclei (Hoechst 33342, blue). The phenotype of GBM (**D, F, and H**) and microglia (**E, G, and I**) was investigated in terms of maximum migration distance, invasion area, and the cell length to width ratio (*N* indicates the total number of ROI, *n* indicates the total number of total cells tested, ** *p* < 0.01, *** *p* < 0.001). Bar charts are expressed as mean ± S.D. Box-and-whisker plots show all individual points on the box.

Since 2D monoculture systems have limitations in the accurate understanding of complex biological systems, it is essential to regulate the spatial and temporal conditions of the defined microenvironment in interaction studies involving the tumor and its TME. Thus, we used a microfluidic 3D GBM TME model to explore the anti-tumor effect of miR-124 EVs on GBM and microglia by co-culturing both cells in the same microenvironment. Although many studies have reported the association of GBM or microglia with miRNAs, there are no reports about the functional demonstration of miRNAs in the complex GBM-microglia microenvironment. We used a PDMS-based 3D microfluidic device to validate whether miR-124 EVs could regulate the progression of GBM while interacting with microglia. This device was also useful in validating the simultaneous and synergistic anti-cancer effects in the complex TME. To determine whether miR-124 EVs had the potential to inhibit tumor metastasis, the migration-inhibiting effect of the EVs on the U373MG cells was assessed. Type-1 collagen was used as a scaffold material in the central channel of the device, and U373MG cells were cultured on one side channel, and microglia on the opposite side channel (Figure 5A). These cells interacted via cytokines/chemokines released through the gel. Using immunocytochemistry, we confirmed that U373MG and microglia were seeded and well separated on both sides of the channels. U373MG cells were stained with anti-S100β and microglia with anti-Iba-1(Figure S9A). On day 4 of the U373MG-microglia co-culture, we examined the migration distance, invasion area, and phenotypical cell characteristics by analyzing images acquired from the 3D microfluidic devices. Based on fully stitched images of the microfluidic device, we observed that the cells invaded into the gel and migrated toward the other cells (Figure S9B). While the presence of miR-NC EVs showed a high level of cell migration, the migration of GBM and microglia was suppressed upon miR-124 EV treatment (Figure 5B-5C). The maximum migrated distance of cells toward the gel was much shorter in the miR-124-treated systems compared to those treated with miR-NC. The same tendency was observed for both GBM and microglia. The results were supported by the earlier results (Figure 2C-2H) showing that miR-124 EVs suppressed the proliferation, migration, and EMT progression in GBMs. For GBM cells, the maximum migration distance was reduced by 27% (*p* < 0.001) upon miR-124 EV treatment (Figure 5D). For microglia, the maximum migration distance was reduced by 31% (*p* < 0.001) (Figure 5E). The average invaded area by cells was reduced by 89% (*p* < 0.001) (Figure 5F) for GBM and 75% (*p* < 0.01) for microglia upon miR-124 EV treatment (Figure 5G). We also examined the change in cell morphological characteristics. Cells that actively migrated tended to have a more elongated phenotype. We measured cell length between the furthest points and the width between the widest points perpendicular to the length. Although the ratio of cell length to width was widely distributed, we observed a significant difference between miR-NC EV- and miR-124 EV-treated groups for both GBM and microglia. Based on average values, GBM treated with miR-124 EVs showed a reduced elongation by 0.62 folds (*p* < 0.001) (Figure 5H), and microglia with miR-124 EVs showed a reduced elongation by 0.43 folds (*p* < 0.001), compared to the miR-NC EV group (Figure 5I). Based on these results, miR-124 EVs significantly affected migration, invasion, and phenotype of both GBM and microglia. MiR-124 EVs had anti-proliferative and anti-migratory effects in both GBM and microglia, meaning that miR-124 EVs could provide an enhanced therapeutic opportunity for the treatment of GBM even in the *in vivo*-like TME.

### Effect of miRNA-124 EVs on patient-derived GBM cells

For the clinical applications of miR-124 EVs, the validation of the therapeutic effects of miR-124 EVs on patient-derived GBM cell lines is required. Therefore, we examined these on five different patient-derived GBM cell lines (SNU-201, SNU-466, SNU-489, SNU-626, and SNU-1105). In microfluidic device experiments (Figure S10-S11), among the five patient-derived cell lines, three lines (SNU-489, SNU-1105, and SNU-201) showed the reduced cell migration and invasion (Figure S10), and two lines (SNU-466 and SNU-626) showed no migration toward the collagen gel, regardless of EV treatment (data not shown). Especially, in microfluidic experiments, SNU-489 cells had very active invasive and migratory characteristics toward the collagen gel, and the invasion area was much larger than those of other cell lines (Figure S10-S11). However, the migration and invasion of the cells were decreased upon miR-124 EVs treatment, which also showed the most improved effects on genes related to anti-tumorigenesis (Figure S12). SNU-489 cell line had a lower number of gene alterations identified than any other cell line as shown in Table S1 56, 57. RT-PCR results revealed that not all genes were regulated but miR-124 EVs showed substantial effects on gene regulation regarding the cell proliferation (*c-Myc*), migration (*ITG-β1*), and EMT (*ZO-1* and *Vimentin*). Expression levels of markers varied according to the GBM cell lines, but the miR-124 EVs had a positive effect of inversely regulating the tumorigenesis in cells. GBM cell lines are known to carry a combination of genetic alterations on tumor suppressors such as *p53*, *p16*, *p15*, *PTEN*, *FHIT*, and *DCC* 58-61. In this study, there were no noticeable correlations between the discovered gene alteration in patient-derived GBM cell lines and their therapeutic responses upon miR-124 EVs treatment. An in-depth study of genes and proteins linked to miR-124 EVs should be investigated further. Nevertheless, the results strongly implied that miR-124 EVs had anti-cancer effects even on heterogeneous subtypes of GBM cells.

### MiR-124 EVs caused a change in the cytokine release in GBM and microglia co-culture

**Figure 6:**
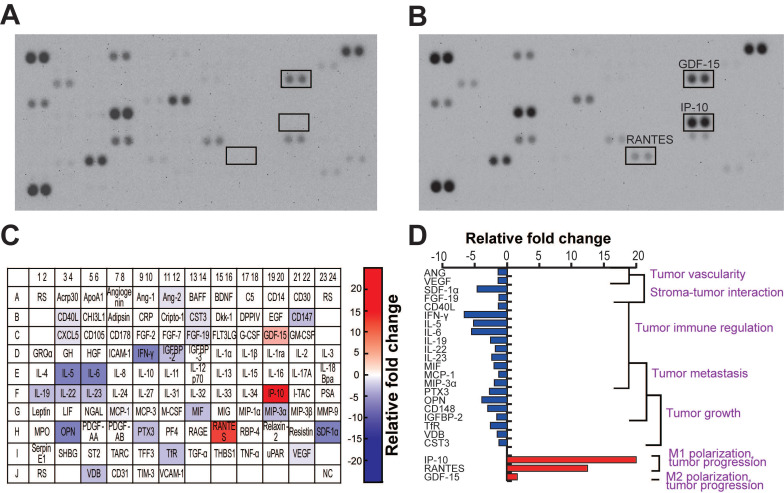
** Cytokine profile in GBM and microglia co-culture in the presence of miR-124 EVs in a microfluidic device.** The cell culture supernatant from day 4 of the co-culture was sampled and analyzed. **(A)** The blots of the miR-NC EV- and (**B**) miR-124 EV-treated devices. **(C)** Schematic representation of the cytokine/chemokine spot positions. Upregulated spots have a red background, and downregulated spots have a blue background. Four levels of the color gradient were set according to the following four different degrees of relative fold changes: 1.25-1.5 folds; 1.5-3 folds; 3-6 folds; 6 folds or more. **(D)** Graphical representation of the relative cytokine levels categorized by functions. ANG, angiopoietin-2; CD40L, CD40 ligand; CST3, cystatin C; FGF-19, fibroblast growth factor-19; GDF-15, growth/differentiation factor-15, IFN-γ, interferon-gamma; IGFBP-2, insulin-like growth factor binding protein-2; IL-5, interleukin-5; IL-6, interleukin-6; IL-19, interleukin-19; IL-22, interleukin-22; IL-23, interleukin-23; IP-10, interferon-gamma-inducible protein-10; MCP-1, monocyte chemoattractant protein-1; MIF, macrophage migration inhibitory factor; MIP-3α, macrophage inflammatory protein-3α; OPN, osteopontin; PTX3, pentraxin3; RANTES, regulated upon activation, normal T cell expressed and presumably secreted; SDF-1α, stromal cell-derived factor-1; TfR, transferrin receptor protein; VDB, vitamin D-binding protein; VEGF, vascular endothelial growth factor.

The TME is very complex and highly associated with various cells, including cancer cells, neighboring stromal cells, and various immune cells, which actively communicate with each other to regulate or promote tumor growth and metastasis. Soluble molecules secreted by glioma cells attract these neighboring cells to the TME, and these cells, in turn, secret cytokines/chemokines that affect the TME 5, 46, 47. To investigate the cytokines released by U373MG and microglia in the same microenvironment, we performed a cytokine profiling of cells co-cultured in the microfluidic device. During the co-culture, cells were treated with miR-NC or miR-124 EVs diluted in the EV-free FBS-containing culture medium for 4 days. We observed that 24 cytokines were differentially regulated by miR-124 EVs compared to miR-NC EVs (Figure 6A-6B). Of these, 21 cytokines were downregulated and 3 cytokines were upregulated in miR-124 EV-treated cells. The results are represented in a color map (Figure 6C). The red and blue background color represents upregulation and downregulation of cytokines, respectively, and the gradient background color implies different cytokine expression levels according to the degree of relative fold changes. We also showed a graphical representation of the relative expression of cytokines categorized by their functions (Figure 6D). Downregulated cytokines were mostly linked to tumor progression factors—tumor growth, metastasis, immune regulation, stroma-tumor interaction, and tumor vascularity. The three upregulated cytokines were also related to tumor progression; however, they were also factors for microglial polarization 4, 9. IP-10 and RANTES, which are regarded as M1 polarization markers 62, were upregulated by more than 10 folds. The slightly upregulated cytokine GDF-15, although not significant, is regarded as an M2 polarization marker 63. Given that a single cytokine may be associated with different roles in the TME, future investigation is needed to clarify the function of the cytokines in the GBM TME. However, it was apparent that tumor-progression-associated cytokines were overall downregulated, and the upregulation of M1 polarization-related cytokines was induced by miR-124 EVs.

### GBM, microglia, and NK cell tri-culture in a 3D microfluidic system

**Figure 7:**
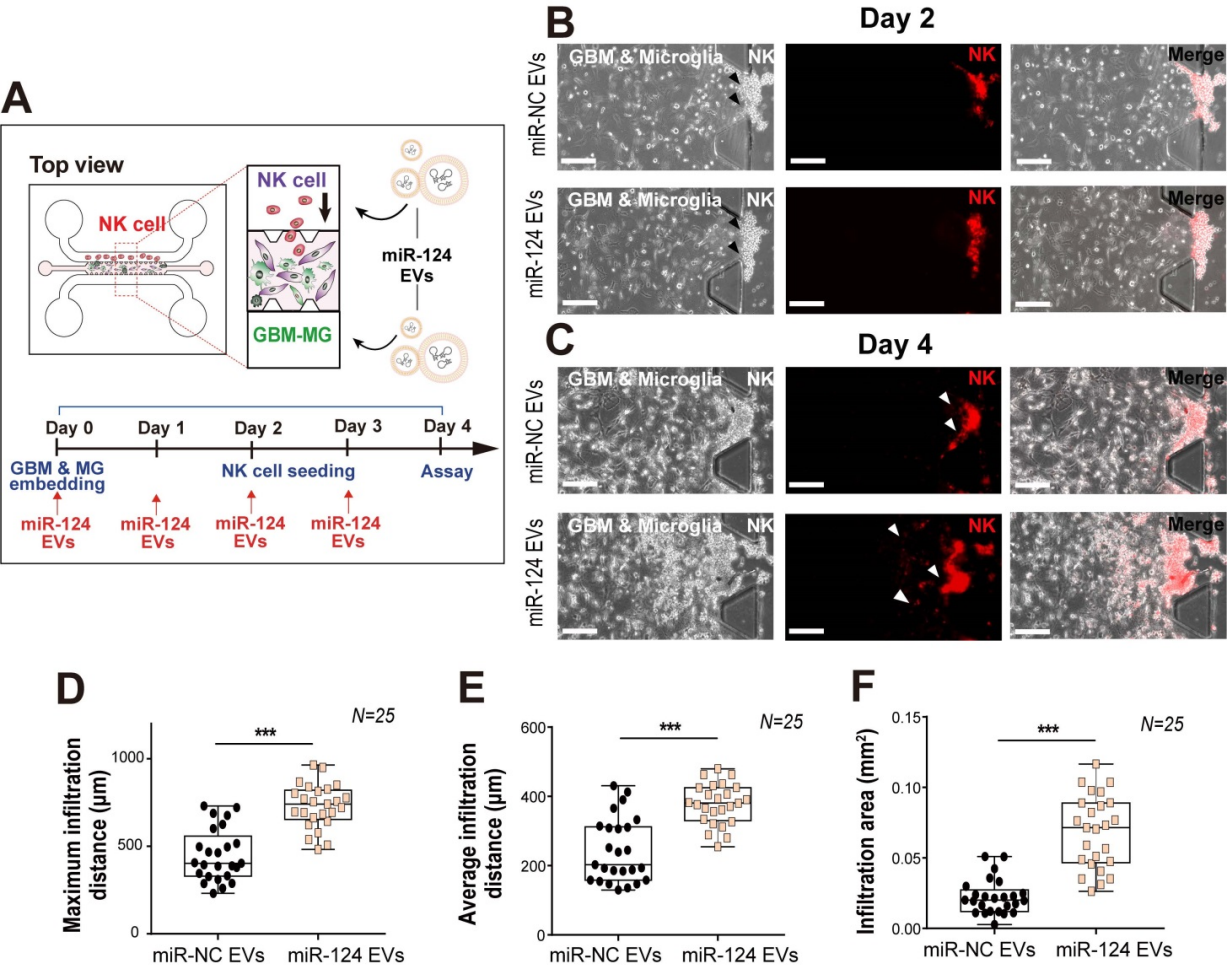
** Intratumoral infiltration of NK cells, induced by miR-124 EVs in a tri-culture system. (A)** U373MG and microglia were embedded in a collagen gel at a ratio of 2:1, 2 days before seeding of NK cells. Representative images of NK cells on (**B**) day 2 and (**C**) day 4 in the microfluidic device treated with miR-NC EVs or miR-124 EVs. Live NK cells were immunostained with PE-conjugated CD 45 (red). **(D)** Maximum infiltration distance, (**E**) average infiltration distance, and (**F**) infiltration area of NK cells were investigated. *N* indicates the total number of ROI. Box-and-whisker plots show all individual points on the box.** *p* < 0.01, *** *p* < 0.001. Scale bar represents 200 µm.

Overall, the cytokine levels released from GBM and microglia into the co-culture microenvironment were found to be reduced except for IP-10 and RANTES, which were dramatically increased. Thus, we next focused on RANTES, given its important chemoattractant role for leukocyte homing and NK cell recruitment. Of note, NK cell infiltration has been associated with an improved prognosis in cancer 64. To determine whether the miR-124 EV-modified GBM microenvironment could affect NK cell recruitment, a tri-culture system was used to assess the infiltration level of NK cells. Approximately one-third of the tumor mass is reportedly occupied by macrophages and microglia 65. U373MG cells and microglia were mixed (2:1), embedded in the gel located in the central channel of the device, and cultured for 2 days with miR-124 EVs. Thereafter, NK-92 cells were added to one medium channel and were analyzed after 2 days (Figure 7A). Analysis of the culture revealed that the NK-92 cells invaded into the gel further in the miR-124 EV-treated group than in the miR-NC EV-treated group (Figure 7B-7C). The maximum infiltration distance, average infiltration distance, and infiltration area increased significantly upon miR-124 EV treatment (*p* < 0.001) (Figure 7D-7F). These results imply that miR-124 EVs promote an anti-tumoral effect on GBM and microglia, by altering the profile of cytokines and chemokines secreted, resulting in enhanced NK cell intratumoral infiltration. Nevertheless, the device cannot be used to explore the crosstalk between innate and acquired immune systems, since various immune cell subtypes are involved in this process through complex mechanisms. Thus, in the future, the microfluidic device needs to be further developed by fine-tuning its design to allow better experimental strategies for investigating the effects of miR-124 EVs on the acquired immune system.

## Materials and methods

### MiRNA preparation

The miRNA mimics (miScript miRNA Mimic, QIAGEN, Germany), miRNA-124 (miR-124, MIMAT0000422), miRNA-128 (miR-128, MIMAT0000424), miRNA-let-7c (miR-let-7c, MIMAT0000064), miRNA-34a (MIMAT0000255), and miRNA-negative control (miR-NC, cat# 1027180), were used in this study.

### Cell culture

Human embryonic kidney 293T (HEK293T) cells and human glioblastoma cell lines U373MG and U87MG (Korean Cell Line Bank, South Korea) were cultured in the Dulbecco's Modified Eagles Medium (DMEM, Lonza, Switzerland) with 10% (v/v) heat-inactivated fetal bovine serum (FBS, Gibco, USA) and 1% (v/v) penicillin/streptomycin (Thermo Fisher Scientific, USA). Human immortalized SV40 microglial cell line (Applied Biological Materials, lnc., USA), derived from primary human microglia, was cultured on collagen-coated flasks (0.15 mg mL^-1^ in 0.02 M acetic acid) in the Prigrow III medium (Applied Biological Materials, lnc., USA) supplemented with 10% FBS (Gibco, USA) and 1% (v/v) penicillin/streptomycin according to the manufacturer's instructions. The human NK cell line NK-92 was purchased from American Type Culture Collection (ATCC, USA). NK-92 cells were grown in the alpha minimum essential medium which contained 12.5% FBS and 12.5% horse serum. To prepare the complete growth medium, 200 U mL^-1^ recombinant IL-2 (Peprotech, USA), 0.2 mM inositol, 0.1 mM 2-mercaptoethanol, and 0.02 mM folic acid were added to the medium before use. Five patient-derived GBM cell lines (SNU-201, SNU-466, SNU-489, SNU-626, and SNU-1105) were purchased from the Korean Cell Line Bank at the Seoul National University. These cells were isolated and established from 54-61 years old Korean male GBM patients. Clinical and genetic characteristics of GBM patient cell lines are summarized in Table S1. Cells were cultured in the RPMI-1640 (Lonza, Switzerland) with 10% (v/v) heat-inactivated FBS (Gibco, USA) and 1% (v/v) penicillin/streptomycin.

### EV preparation

For the carrier EV preparation and its subsequent miRNA loading, we followed previously reported protocols 27, 66, 67. Briefly, HEK293T cells were cultured in media supplemented with EV-free FBS. The EVs were isolated from 160 mL of the conditioned media using ExoQuick-TC^TM^ (System Biosciences, USA) according to the manufacturer's instructions. Respective miRNAs were loaded onto the harvested carrier EVs, followed by ultrafiltration (UF) after miRNA loading as described in the next section.

To prepare GBM EVs, U373MG conditioned media were collected from confluent U373MG cells cultured in two T75 flasks. Cells were cultured in media supplemented with EV-free FBS for 24 h, and GBM EVs were isolated using ExoQuick-TC^TM^ according to the manufacturer's instructions. Briefly, U373MG-conditioned media (20 mL) were centrifuged for 15 min at 1500 × *g* to remove cell debris. The final isolated GBM EV pellet was resuspended in 100 μL PBS (approximately 3×10^10^ particles mL^-1^) and then diluted to 3×10^9^ particles mL^-1^ in culture media containing 10% (v/v) EV-free FBS for the stimulation of microglial cells. To calculate the amount of miRNA to load into the EVs, a particle concentration-based unit was used via NTA analysis instead of a protein concentration-based unit because EV-containing solution analyzed using a protein assay does not reflect the exact amount of EVs due to protein contamination 48.

### Preparation of miRNA-loaded EVs

The harvested carrier EVs were used to load miRNA mimics using the Lipofectamine RNAiMAX kit (Invitrogen, USA) according to the manufacturer's instructions and a previous report 27. Briefly, 800 pmol of miRNAs were mixed with the reagent and incubated with 2×10^12^ EVs for 6 h at 37 °C. The mixture was ultra-filtered with a 100-kDa filter (Merck Millipore, USA) to remove free unloaded miRNAs. The final stock concentration of miRNA EVs was 1×10^13^ particles mL^-1^. The quality of EVs, such as loading efficiency or stability, was assumed to be identical to those prepared in the previous report 27.

### NTA and zeta potential analysis

The size and concentration distributions of the EVs were analyzed using the NanoSight NS300 (Malvern, UK) equipped with the NTA software (Version 3.2, Malvern, UK). The zeta potential of the EVs was analyzed using the Zetasizer Nano ZS (Malvern, UK) equipped with the Zetasizer software (Version 7.12, Malvern, UK).

### TEM analysis

TEM analysis was used to image and characterize the EVs before and after miRNA loading. The harvested carrier EVs or miRNA EVs were diluted in PBS and absorbed onto a carbon-coated grid, and they were negatively stained with 2% uranyl acetate for 1 min. The samples were dried and observed using the JEM-1400plus electron microscope (JEOL, Japan) at the Korea Basic Science Institute in South Korea.

### Cell proliferation and viability assays

Proliferation and viability were analyzed using the Cell Counting Kit-8 (CCK-8, Dongin LS, South Korea) assay according to the manufacturer's instructions. U373MG or U87MG cells were seeded in 96-well plates (5×10^4^ cells mL^-1^ and 1×10^5^ cells mL^-1^ in each well, respectively). The assay was performed 72 h after miRNA lipofection (Lipofectamine RNAiMAX kit, Invitrogen, USA) or miRNA EV treatment. The absorbance was measured using a microplate reader (DTX880, Beckman Coulter, USA) at 450 nm.

### qRT-PCR

U373MG and microglial cells were cultured for 48 h under lipofection or miRNA EV treatment. Subsequently, GBM EVs were added to the microglial cells for an additional 18 h. Total mRNA was extracted from the cells using the RNeasy^®^mini kit (QIAGEN, Germany) and RNA yields were measured using the nanophotometer™ (P330, IMPLEN, Germany). Total mRNA (100-300 ng) was reverse-transcribed using the ReverTra Ace® qPCR RT Master Mix with the gDNA Remover kit (Toyobo Co., Japan), and cDNA (1-2 µL) was mixed with the qPCR master mix (Power SYBR® Green PCR Master Mix, Applied Biosystems, USA). As part of miRNA quantification, the miScript RT II kit (Qiagen, Germany) and the miScript SYBR Green PCR kit (Qiagen, Germany) were used for cDNA synthesis and to prepare the qPCR master mix, respectively. The samples were subjected to 40 amplification cycles using a real-time PCR thermal cycler (QuantStudio 3, Applied Biosystems, USA). In U373MG, proliferation markers *c-Myc* and *Mcl-1*, migration-associated marker integrin-β1 (*ITG-β1*), epithelial marker *ZO-1*, and mesenchymal markers *Slug* and *Vimentin* were examined. In microglia, M1 (*TNF-α*, *IL-6*, and *NOS2*) and M2 (*TGF-β*, *IL-10*, and *ARG1*) phenotypic markers were examined to classify their polarization status. Signal transducer and activator of transcription *(STAT) 1* and *STAT3* expression in both U373MG and microglial cells were also examined. GAPDH was used as an internal control. The sequences of primers used are listed in Table S2. For patient-derived GBM cell lines, the same sequenced primers were used in RT-PCR experiments.

### TMZ treatment

TMZ stock was diluted to 100 mM with DMSO. The survival of U373MG cells was assessed via a CCK-8 assay after treatment with temozolomide (25-400 μM, Sigma-Aldrich, USA) in the culture medium for 48 h. The final concentration of DMSO did not exceed 0.4% (v/v) in the culture medium.

### Western blot analysis

For western blot analysis, both U373MG and microglial cells treated with miR-124 EVs (1×10^12^ particles mL^-1^) were lysed in the RIPA lysis buffer (Thermo Fisher Scientific, USA) with protease inhibitor cocktail (Roche, Switzerland) and phosphatase inhibitor cocktail (Thermo Fisher Scientific, USA). The concentration of the lysed proteins was determined using the BCA protein assay kit (Thermo Fisher Scientific, USA) according to the manufacturer's instructions. The protein lysate (10-30 μg) was loaded in each well of the Bolt™ 4-12% Bis-Tris Plus Gel (Invitrogen, USA) and electro-transferred onto a PVDF membrane (GE Healthcare, USA). Primary antibodies used were anti-STAT3 (1:1000, Cell Signaling Technology USA) and anti-pSTAT3 (1:2000, Cell Signaling Technology USA). Anti-GAPDH (1:200, Santa Cruz Biotechnology, USA) was used as an internal control. The membrane was rinsed and incubated with HRP-conjugated secondary antibodies (1: 2000) for 1 h at 25 °C. The membrane was rinsed and ECL solution (GE Healthcare, USA) was used to detect chemiluminescent signals using a gel imaging system (ImageQuant 350, GE Healthcare, USA). For the analysis of the carrier EVs derived from HEK293T cells, primary antibodies anti-CD63 (1:2000, System Biosciences, USA) and CD81 (1: 1000, Abcam, UK) were used as EV markers. The overall process was the same as described above.

### Microfluidic device fabrication

The microfluidic chip consisted of one gel channel at the center and two medium channels on both sides. The trapezoidal-shaped micropillars were arrayed along the gel channel on both sides with a regular gap distance (200 µm) between them. The widths of the gel channel and medium channel were 1.3 and 1 mm, respectively. The total length of the channels was about 15 mm, and their depth was about 200 µm. The overall fabrication process of the 3D cell culture device followed a standard soft lithography protocol according to our previous reports 27, 68. After fabrication of Si master mold, the Sylgard 184 elastomer (Dow Corning, USA), poly-dimethylsiloxane (PDMS) with a curing agent was poured on the mold to a thickness of about 3 to 4 mm and baked at 80 °C for 2 h for polymerization. The PDMS replica was peeled off and the inlet and outlet holes (4 mm diameter) were punched out to connect to gel and medium channels. The PDMS layer was attached to the cover glass (24 × 24 mm) via plasma treatment. PDMS channels were coated with poly-D-lysine hydrobromide (PDL, 1 mg mL^-1^, Sigma-Aldrich, USA) for 4 h to increase the electrostatic interactions between the gel and channel surfaces. The channels were rinsed and the microfluidic devices were dried in an oven at 80 °C.

### GBM-microglia co-culture in a 3D microfluidic device

GBM and microglial cells were co-cultured in a 3D microfluidic device. First, type I collagen (Corning, USA) was prepared following the protocol described in our previous report 27. Collagen solution (2 mg mL^-1^ Corning, USA) was injected through the central gel channel and incubated for 30 min at 37 ºC. After gelation, a suspension of U373MG (1×10^6^ cells mL^-1^, 40 μL) was introduced into one side of the medium channel. Owing to the flow induced by the hydraulic pressure across the gel, the cells were instantly attracted to the gel channel and attached to the sidewalls of the collagen gel. After seeding, the device was placed in an incubator at 37 ºC for 1 h for stabilization. Sequentially, a suspension of microglia (1×10^6^ cells mL^-1^) was also introduced to the opposite side of the medium channel under hydraulic pressure. Microglial cells were attached to the opposite sidewalls of the collagen gel. Each channel was supplied with the culture medium for U373MG and microglia, respectively. MiRNA EVs mixed in the culture medium were added to both cell channels on the day of the seeding. The media in the cell channels were replaced every day with media that contained miRNA EVs (1×10^12^ particles mL^-1^). Half the volume of original media was removed and refilled. Phenotypical changes, cell migration, and invasion of both cells were monitored and assayed on day 3 or 4 of the co-culture. In the experiments with patient-derived GBM cell lines, the assays were performed on day 3 of the co-culture. Other experiments were the same as described above.

### Cytokine profiling

U373MG and microglial cells were co-cultured in a 3D microfluidic device and cytokine profiling was performed using a human cytokine antibody array kit (Proteome profiler human XL cytokine array kit, R&D Systems, USA). Cell culture supernatants were collected from independent microfluidic devices on day 4 of the co-culture. For detection, 350 μl of the cell culture supernatants, pooled from microfluidic devices on day 4, was used, and the assay was performed according to the manufacturer's instructions. Briefly, after centrifugation of supernatants, the membrane was incubated with supernatants to assess the levels of 105 cytokines. Immunoblotting images were visualized and captured using a gel imaging system (ImageQuant 350, GE Healthcare, USA), and the intensities of each spot were analyzed using the Image J software.

### Glioblastoma-microglia-NK cell tri-culture in a 3D microfluidic device

U373MG and microglia (2:1) were mixed in type I collagen solution (final 2 mg mL^-1^) and diluted to 5×10^5^ cells mL^-1^. The gel solution was injected through the central gel channel and incubated for 30 min at 37 °C for gelation. The 1:1 mixed culture medium for U373MG and microglia containing miRNA EVs was supplied to the medium channel for 2 days. Then, a suspension of NK cells (1×10^6^ cells mL^-1^, 40 μL) was introduced into one side of the medium channel to attach the cells to the gel side. The tri-culture was maintained for another 2 days with miRNA EVs. The channels were replaced every day with a medium that contained the miRNA EVs. The infiltration of NK cells into the U373MG-microglia area was analyzed on day 2 after tri-culture.

### Immunostaining

For the immunostaining of cells in the 3D microfluidic device, the cells were fixed with 4% (v/v) paraformaldehyde for 20 min at 25 °C and permeabilized with 0.1% (v/v) Triton X-100 in DPBS for 20 min. Actin filaments and nuclei were stained with phalloidin (1:40, Sigma-Aldrich, USA) and Hoechst 33342 (1:2000, Thermo Fisher Scientific, USA). During the co-culture of GBM and microglia, every region of interest (ROI) was monitored, and fluorescent images were obtained using a fluorescence microscope (THUNDER Imager 3D Live 3 Cell, Leica, Germany). The 3D maximum projection images of 1.84-μm thick Z-stacks (total 146 μm thick) from replicate samples were acquired using the Leica software LAS X (Leica, Germany). Other fluorescence images were acquired using a fluorescence microscope (CELENA X, Logos Biosystems, South Korea). For immunostaining with tumor-specific (S100β) and microglia-specific (Iba-1) antibodies, cells were fixed, permeabilized, and blocked with 3% (w/v) bovine serum albumin (BSA, MP Biomedicals, USA) for 1 h at 25 °C in DBPS containing 0.1% (v/v) Triton X-100. The anti-S100β (1:500, Thermo Fisher Scientific, USA) and anti-Iba-1(1:500, Wako, Japan) primary antibodies diluted in 1% (w/v) BSA containing Tween-20 (Duchefa Biochemie, Netherlands) were used, and Alexa fluor-488 (1:400) and 594 (1:100) conjugated antibodies (Invitrogen, USA) were used as the secondary antibodies. For staining of live NK-92 cells with surface antigen (CD45), cells were mixed with a solution containing PE-conjugated CD45 antibody (Beckman Coulter, USA) for 15 min before use.

### Image analysis

The replicated projection images were analyzed quantitatively using the Image J software. For the experiments on the migration and invasion analysis of U373MG, patient-derived GBM cell lines, and microglia, 14-15 ROI images were acquired for each condition, and all binary and thresholded images were analyzed. The proportion of the fluorescence pixels was calculated for every image. To assess the level of elongation of the cells, we measured the cell length and width in 15 ROI images, and the number of measured cells was 74 and 21 for U373MG and microglia, respectively. For the NK cell analysis, 25 ROI images were acquired for each condition. Cytokine array experimental data were also analyzed using the Image J software, and the intensity of every dot was measured.

### Statistical analysis

All experiments were conducted in at least triplicates. Bar charts are expressed as the mean ± S.D. Box-and-whisker plots show all individual points on the box. The significance of the difference between two independent groups was determined using a two-tailed Student's *t*-test. Differences were considered to be statistically significant at * (*p* < 0.05), ** (*p* < 0.01), and *** (*p* < 0.001).

## Conclusions

GBM is the most common and aggressive cancer in the brain. The relationship between GBM and microglia has been consistently noted by researchers as it provides a potential clue for understanding TME. The complexity of TME poses various challenges that ought to be surmounted in developing more effective therapeutic strategies. A miRNA-based EV drug is one of the potential candidates to overcome these issues. In particular, EV drugs have a unique advantage, such as the superior potential of BBB penetration. In this study, we demonstrated the potential therapeutic effects of miR-124 EVs in the environment where GBM and microglia interact. We revealed that miR-124 delivered via EVs has a suppressive effect on the proliferation and metastatic characteristics of GBM via STAT3 regulation. MiR-124 also disturbed the M2 polarization of microglia by targeting STAT3. We evaluated miR-124 EV-based therapeutics in more complex GBM TME models *in vitro* using a 3D microfluidic device. Analyses of cytokines released by the TME model demonstrated that miR-124 EVs successfully regulate the growth, migration, and metastasis of tumors, and create a tumor-suppressive and immune-favorable microenvironment. Based on the results, the key advantage of our miR-124 EVs is that their therapeutic effects are exerted by simultaneously targeting both cancer cells and microglia. In conclusion, we suggest through concrete evidence that the EV-based miRNA delivery combined with the microfluidic technology will contribute to further understanding of the GBM microenvironment involving tumor and immune cells, and will promote the development of potential therapeutic strategies using miRNA-loaded EVs.

## Supplementary Material

Supplementary figures and tables.

## Abbreviations

ANG: angiopoietin-2
ARG1: arginase 1
CCK-8: Cell Counting Kit- 8
CD40L: CD40 ligand
c-Myc: c-myelocytomatosis
CST3: cystatin C
EMT: epithelial-mesenchymal transition
EVs: extracellular vesicles
EXQ: ExoQuick method
FGF-19: fibroblast growth factor-19
GBM: glioblastoma multiforme
GDF-15: Growth/differentiation factor-15
HEK293T: human embryonic kidney 293T
IFN-γ: interferon-gamma
IGFBP-2: insulin-like growth factor binding protein-2
IL-5: interleukin-5
IL-6: interleukin-6
IL-10: interleukin-10
IL-19: interleukin-19
IL-22: interleukin-22
IL-23: interleukin-23
IP-10: interferon-gamma inducible protein-10
ITG-β1: integrin beta-1
JAK: janus-activated kinase
Mcl-1: myeloid cell leukemia-1
MCP-1: monocyte chemoattractant protein-1
MIF: macrophage migration inhibitory factor
MIP-3α: macrophage inflammatory protein-3α
miR-124 Evs: microRNA-124-loaded EVs
NOS2: nitric oxide synthase 2
NTA: nanoparticle tracking analysis
OPN: osteopontin
PTX3: pentraxin3
RANTES: regulated upon activation, normal T cell expressed and presumably secreted
S100β: S100 calcium-binding protein B
S727: serin 727
SDF-1α: stromal cell-derived factor-1
STAT1: signal transducer and activator of transcription 1
STAT3: signal transducer and activator of transcription 3
TEM: transmission electron microscopy
TfR: transferrin receptor protein
TGF-β: transforming growth factor-β
TME: tumor-microenvironment
TMZ: temozolomide
TNF-α: tumor necrosis factor-α
UF: ultrafiltration
VDB: vitamin D-binding protein
VEGF: vascular endothelial growth factor
Y705: tyrosine 705
ZO-1: zonula occludens-1
