# Supplementary material for: Inhibition of tumor progression and M2 microglial polarization by extracellular vesicle-mediated microRNA-124 in a 3D microfluidic glioblastoma microenvironment
Source: Theranostics. 2021 Sep 27;11(19):9687–704. doi: 10.7150/thno.60851 (PMC8490520; doi:10.7150/thno.60851)
Supplement: Supplementary file 1 — Supplementary figures and tables. [file thnov11p9687s1.pdf]

Supplementary Material

Inhibition of tumor progression and M2 microglial polarization by extracellular vesicle-mediated microRNA-124 in a 3D microfluidic glioblastoma microenvironment

Soohyun Hong, Jae Young You, Kyurim Paek, Jubin Park, Su Jin Kang, Eun Hee Han, Nakwon Choi, Seok Chung, Won Jong Rhee, Jeong Ah Kim**

Table S1. Characteristics of the patient-derived GBM cell line [1, 2].

Cell line	Age(yr)/Sex	Diagnosis	Prior therapy	Nuclear pleomorphism	Inactivation of tumor suppressor genes identified
**SNU-201**	58/M	GBM	None	+	*p53, p16, p15, DCC*
**SNU-466**	61/M	GBM	None	+	*p16, p15, PTEN*
**SNU-489**	54/M	GBM	None	+	*p53, DCC*
**SNU-626**	65/M	GBM	None	+	*p53, PTEN, DCC*
**SNU-1105**	61/M	GBM	None	+	*p53, p16, p15*

Table S2. List of primer sequences used for qRT-PCR.

Gene name	Forward primer sequences	Reverse primer sequences
GAPDH	GTCAGTGGTGGACCTGACCT	CACCACCTGTTGCTGTAGC
c-Myc	GTCAAGAGGCGAACACACAACGT	GGGCCTTTTCATTGTTTTCCAAC
Mcl-1	CCAAGAAAGCTGCATCGAACCAT	CAGCACATTCCTGATGCCACCT
ITG-β1	CAAGCAGGGCCAAATTGTGG	GCAGAAGTAGGCATTCCTTCC
ZO-1	GCGGTCAGAGCCTTCTGATC	CATGCTTTACAGGAGTTGAGACAG
Vimentin	AATGCGTCTCTGGCACGTCTTG	CTTCTGCCTCCTGCAGGTTC
Slug	TTCTCTAGGCCCTGGCTGCTA	CTTGACATCTGAGTGGGTCTGGA
TNF-α	CCACTTCGAAACCTGGGATTC	TTAGTGGTTGCCAGCACTTCA
IL-6	GTCCAGCCTGAGGGCTCTTC	TCTGTGCCCAGTGGACAGGT
NOS2	ACACGCCCACAGAGATCCACCT	TTCCGCACAAAGCAGGGCACT
ARG-1	ACCAGTCGTGGGAGGTCTGACAT	TGTCTTCCCCAGGGATGGGTT
TGF-β	TACCGCTGCTGTGGCTACTGGT	AGCCGCAGCTTGGACAGGATCT
IL-10	GTGATGCCCCAAGCTGAGAACC	AGGCATTCTTCACCTGCTCCAC
STAT1	AGTGGTACGAACTTCAGCAGCT	GACATCTGGATTGGGTCTTCCTG
STAT3	AGCAGCACCTTCAGGATGTCC	GCATCTTCTGCCTGGTCACTG

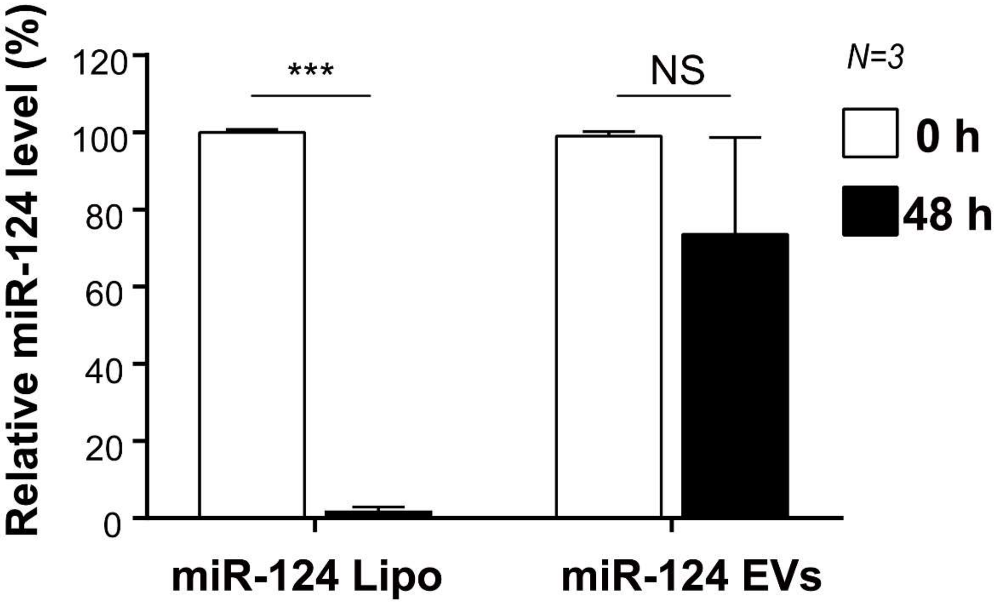

Figure S1. MiRNA stability in EVs. The stability of miR-124 EVs was compared with the same amount of miRNAs loaded in liposomes (using Lipofectamine) without EVs. All samples were incubated at 37 ºC for 48 h and ultrafiltered with a 100-kDa filter and the miR-124 levels were measured using qRT-PCR. All values are expressed as the mean ± S.D. (*** $p < 0.001$; NS, not significant).

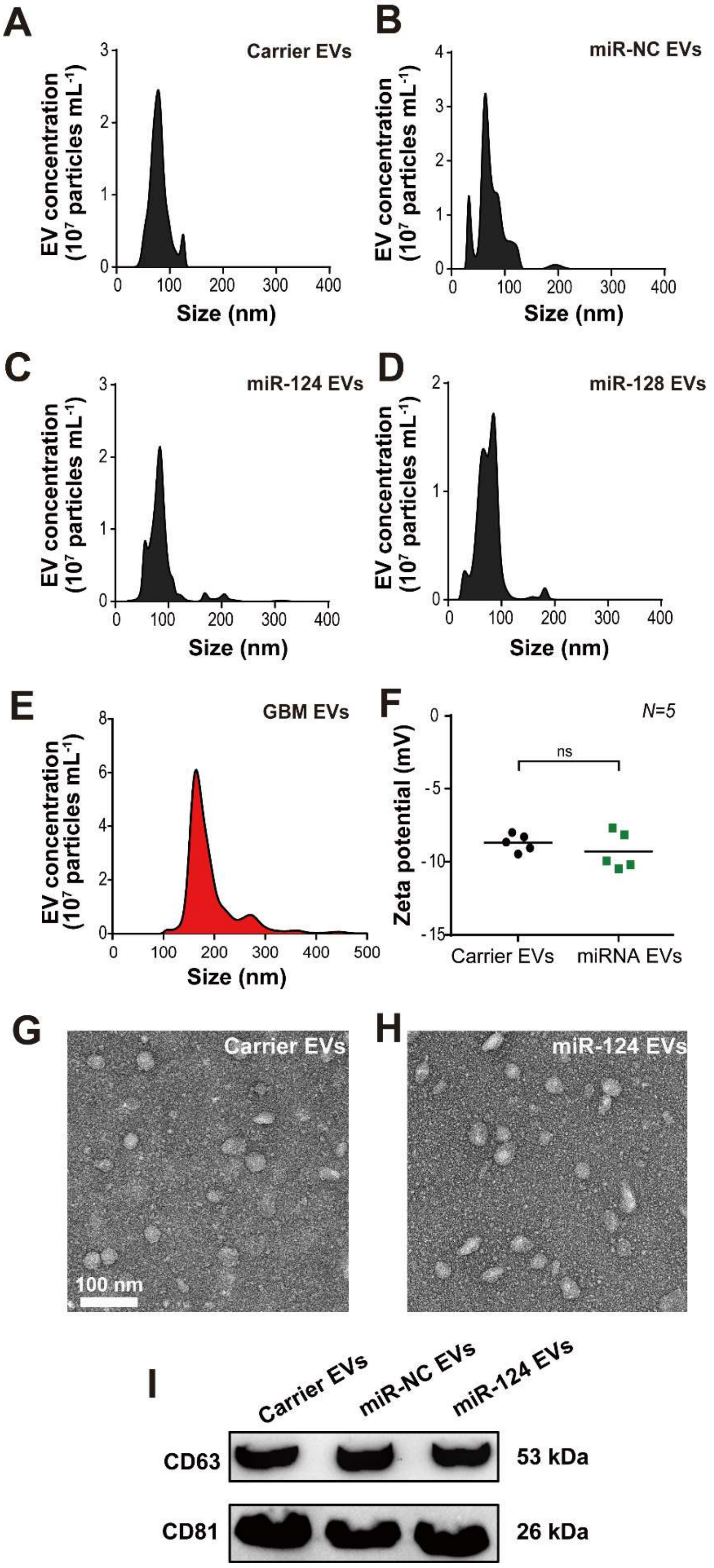

A
Carrier EVs
EV concentration (10⁷ particles mL⁻¹)
Size (nm)
B
miR-NC EVs
C
miR-124 EVs
D
miR-128 EVs
E
GBM EVs
F
N=5
ns
Zeta potential (mV)
Carrier EVs
miRNA EVs
G
Carrier EVs
100 nm
H
miR-124 EVs
I
Carrier EVs
miR-NC EVs
miR-124 EVs
CD63
53 kDa
CD81
26 kDa

Figure S2. EV characterization. The size distribution of HEK293T cell-derived EVs before and after miRNA loading was analyzed using NTA. (A) Carrier EV, (B) miR-NC-EV, (C) miR-124 EV, and (D) miR-128 EV. Carrier EV represents the isolated EV from the HEK293T-conditioned medium using ExoQuick-TC^{TM}. Then, miRNAs were loaded into carrier EVs using a transfection reagent and purified via ultrafiltration to remove unloaded miRNAs. (E) The size distribution of GBM-derived EVs. GBM EVs were isolated from the U373MG cell-conditioned medium using ExoQuick-TC^{TM} and used to activate microglial cells. (F) Zeta potential of EVs before and after miRNA loading. (G and H) TEM images of EVs before and after miRNA loading. (I) Western blot analysis of EVs before and after loading miR-NC or miR-124. CD63 and CD81 are EV markers. (ns, not significant).

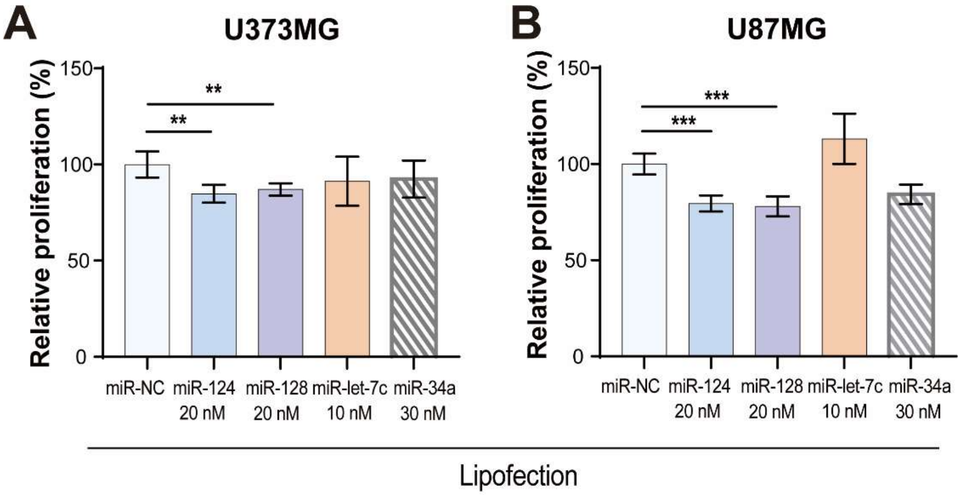

Figure S3. MiRNA candidate screening for anti-proliferation of GBM. Proliferation assay was performed after 72 h of miRNA lipofection in GBM cells using Lipofectamine RNAiMax. All miRNA candidates (miR-124, miR128, miR-let-7c, and miR-34a) were evaluated in (A) U373MG and (B) U87MG cell lines. The relative proliferation rate was compared to miR-NC EVs. Results show the representative efficiency of each miRNA ranging from 10 to 30 nM. All values are expressed as the mean ± S.D. (** $p < 0.01$, *** $p < 0.001$).

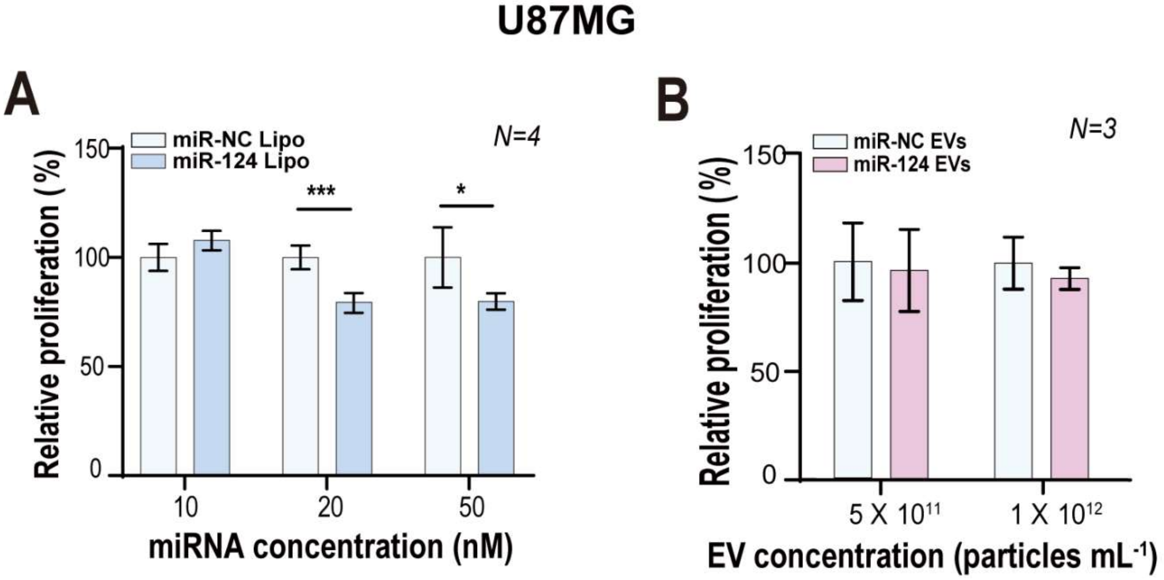

Figure S4. Anti-tumor effect of miR-124 in U98MG cells. (A) The anti-proliferative effect of miR-124 lipofection in U87MG cells. Proliferation assay was performed 72 h after U87MG cells had been transfected with miRNA at 10, 20, and 50 nM. (B) Anti-proliferative effect of miR-124 EVs in U373MG cells. Two different concentrations of EVs (5×10^{11} particles and 1×10^{12} particles per mL) were added to U87MG cells for 72 h. All values are expressed as the mean ± S.D. (* $p < 0.05$, *** $p < 0.001$).

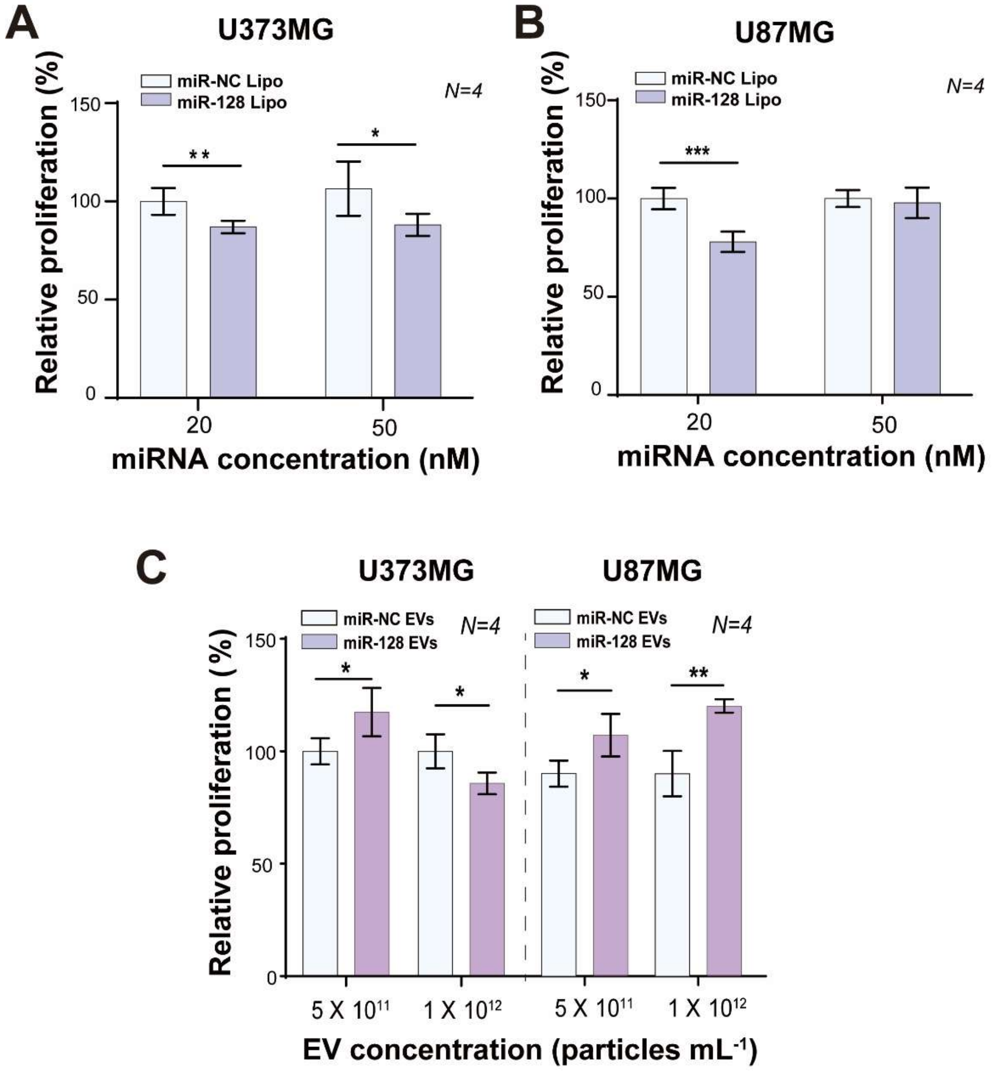

Figure S5. Dose-dependent anti-proliferative effect of miR-128 in GBM. (A) Anti-proliferative effect of miR-128 lipofection in GBM cells, (A) U373MG and (B) U87MG. Proliferation assay was performed after 72 h of miRNA lipofection in GBM cells. The efficiency of 20 nM and 50 nM miRNAs was evaluated. (C) Anti-proliferative effect of miR-128 EVs on GBM cells, U373MG and U87MG. EVs were added at two different concentrations (5×10^{11} particles and 1×10^{12} per particles mL) to both GBM cells for 72 h. All values are expressed as the mean ± S.D. (* $p < 0.05$, ** $p < 0.01$, *** $p < 0.001$).

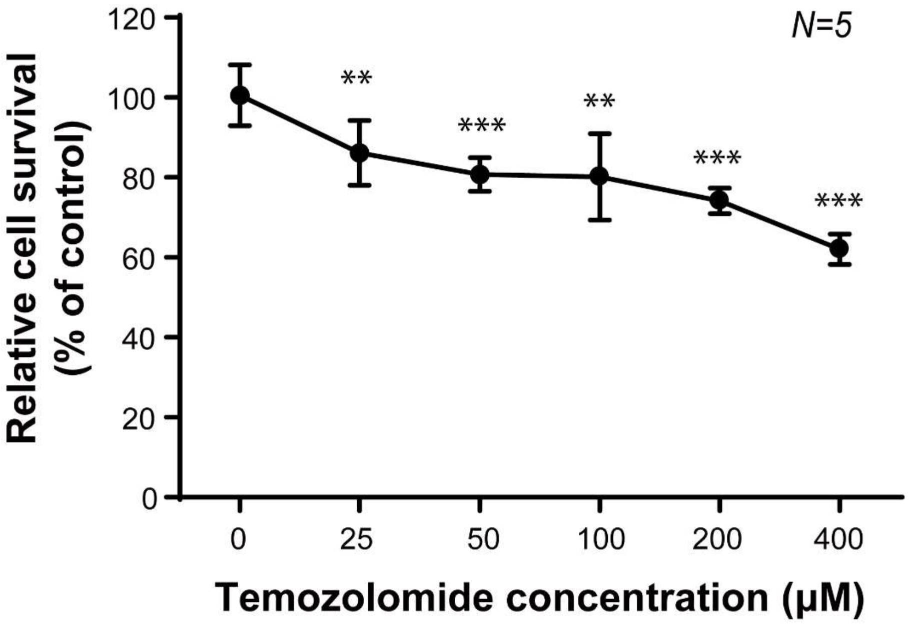

Figure S6. Dose-dependent cell viability of GBM upon temozolomide (TMZ) treatment. Relative cell viability of U373MG was measured at 48 h after TMZ treatment at a concentration range of 25–400 μM. Cell viability was normalized to that of the control group (0.1% DMSO-treated). All numerical data are expressed as the mean ± S.D. (** $p < 0.01$, *** $p < 0.001$).

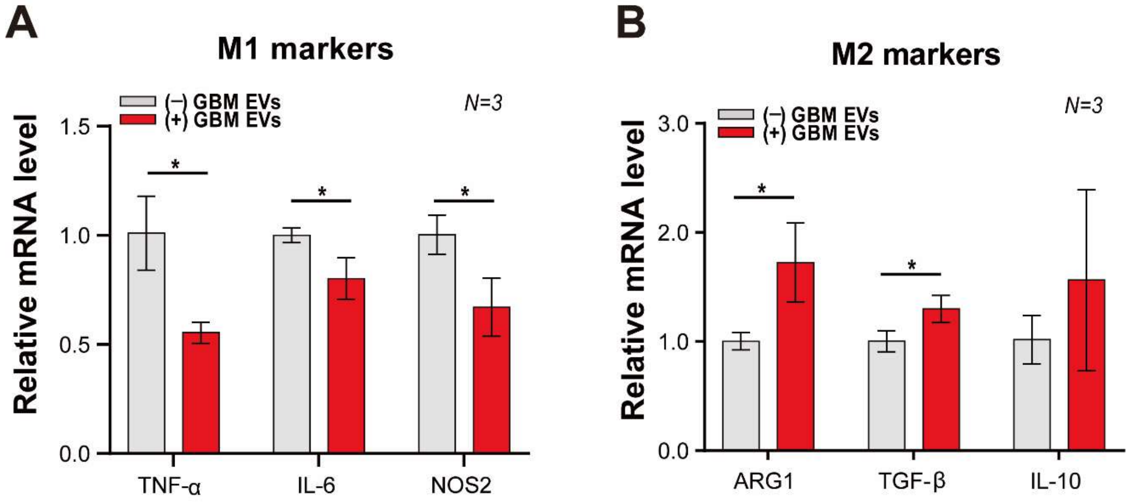

Figure S7. M/M2 polarization in microglia stimulated by GBM-derived EVs. The relative mRNA expression level of (A) M1 and (B) M2 microglial polarization with or without GBM-derived EVs. Microglia were treated with GBM-derived EVs (3×10^9 particles mL^{-1}) for 18 h. GAPDH was used as an internal control. All values are expressed as the mean ± S.D. (* $p < 0.05$).

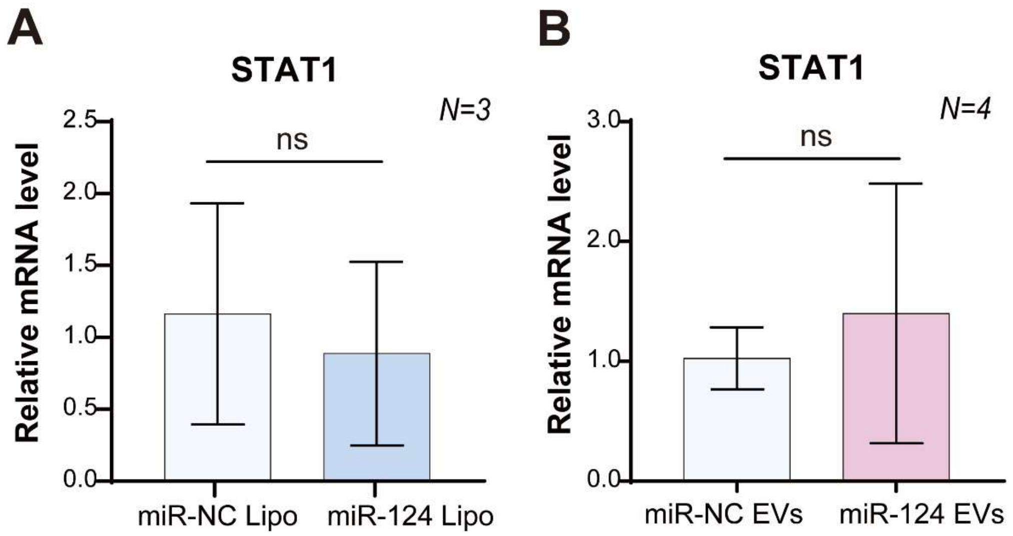

Figure S8. Change in *STAT1* miRNA expression in GBM by miR-124. Relative mRNA expression of *STAT1* in GBM by (A) miR-124 lipofection and (B) miR-124 EV treatment. Cells were transfected with 20 nM miRNAs for 72 h, or with miR-124 EVs (1×10^{12} particles mL^{-1}) for 48 h. All values were normalized to those of the miR-NC-treated group and are expressed as the mean ± S.D. (ns, not significant).

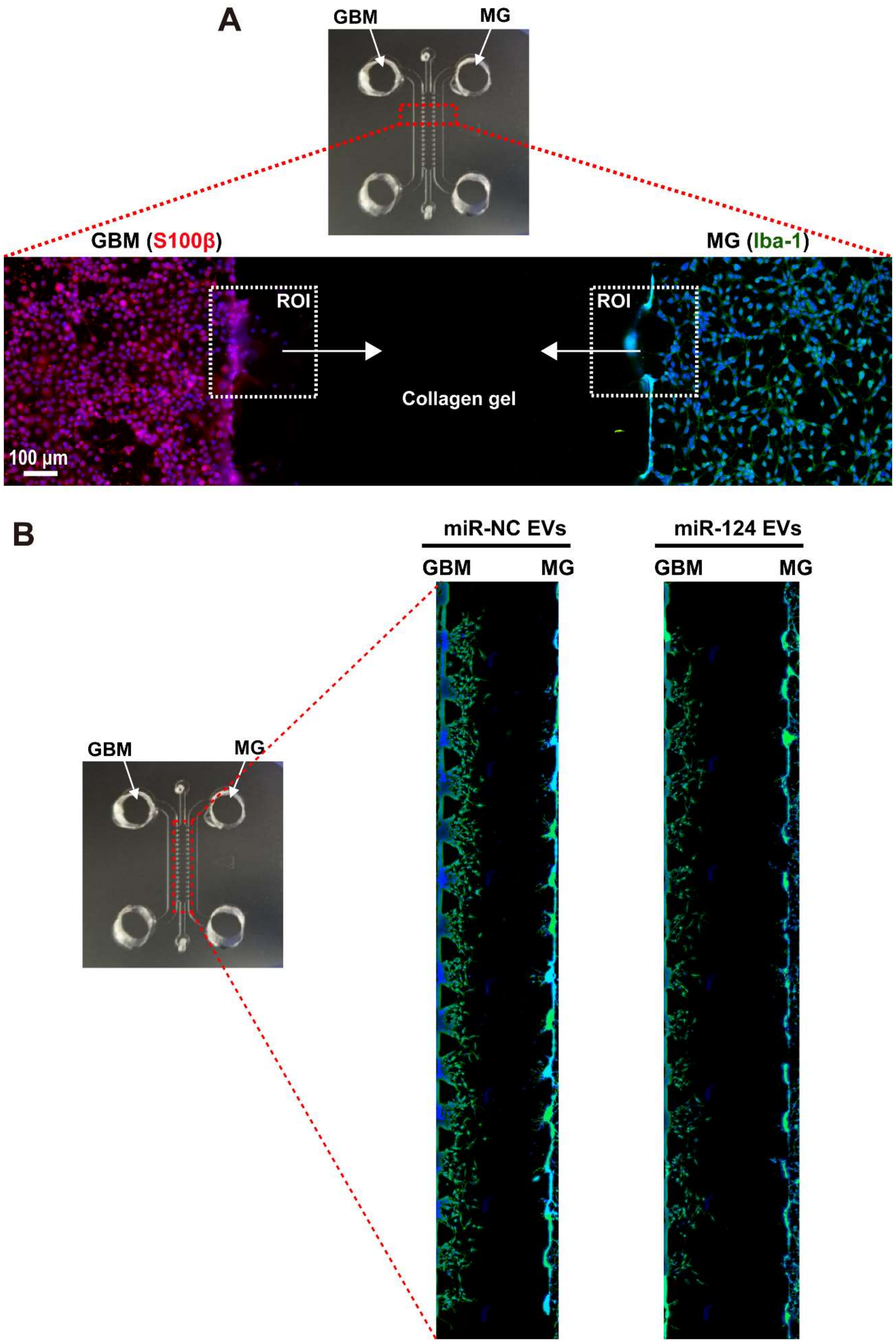
A
GBM
MG
GBM (S100β)
MG (Iba-1)
ROI
ROI
Collagen gel
100 μm
B
miR-NC EVs
miR-124 EVs
GBM
MG
GBM
MG
GBM
MG

Figure S9. Co-culture of GBM and microglia in a 3D microfluidic device. (A) Representative images of immunostained GBM and microglia co-cultured in a microfluidic device. (A) U373MG and microglia were immunostained against S100β (astrocyte marker, red) and Iba-1 (microglial marker, green). (B) U373MG and microglia were co-cultured for 4 days in a microfluidic device with culture medium containing miR-NC EVs or miR-124 EVs (1×10^{12} particles mL^{-1}), and immunostained for F-actin (Phalloidin, green) and nuclei (Hoechst 33342, blue). Fully stitched image showing entire fields of ROIs in the microfluidic device. The 3D maximum projection images with Z-stack were stitched together.

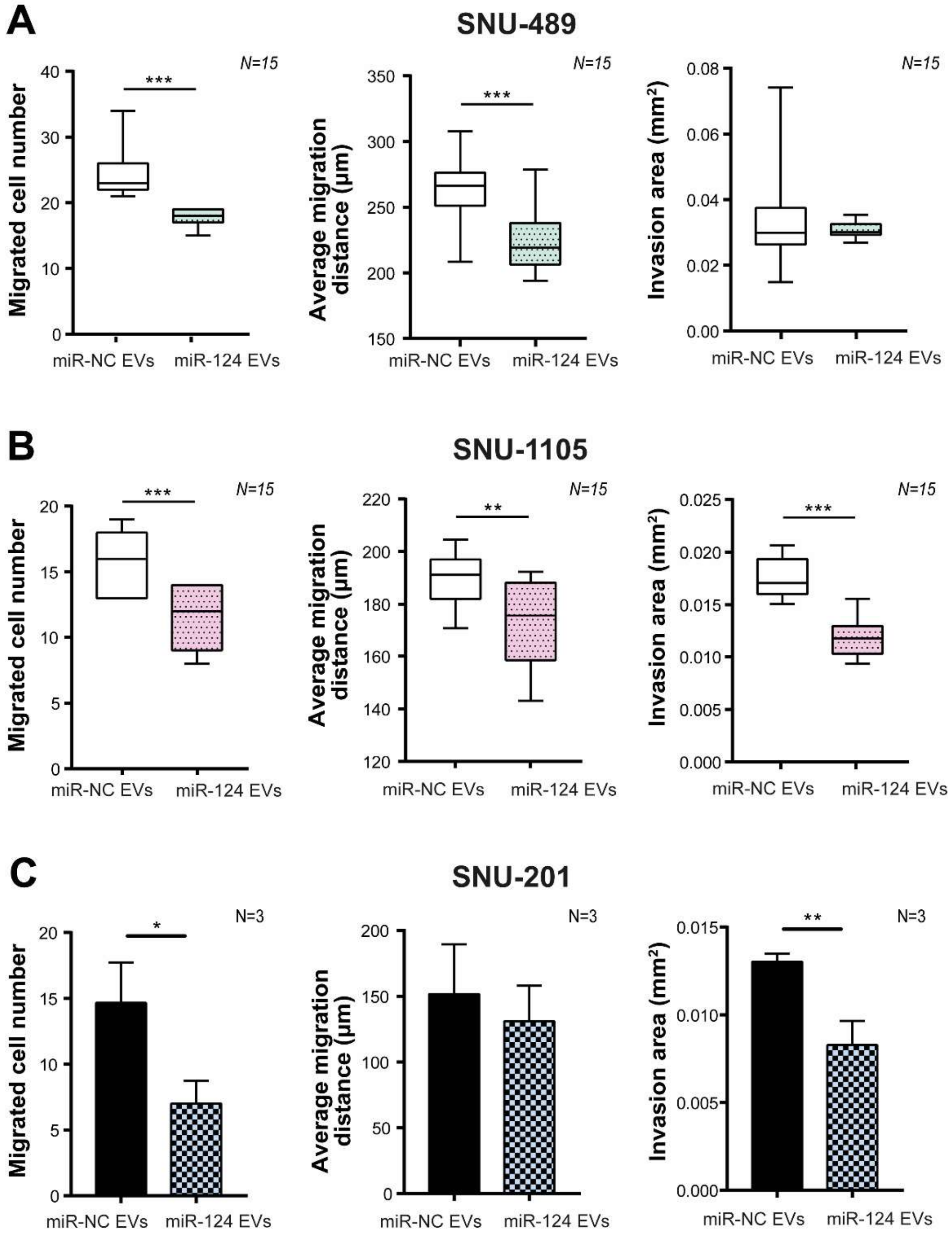

Figure S10. Effect of miR-124 EVs on patient-derived GBM cell lines in a 3D microfluidic device. Each patient-derived GBM cell line, (A) SNU-489, (B) SNU-1105, and (C) SNU-201, was co-cultured with microglia for 3 days in a microfluidic device with culture medium containing miR-NC EVs or miR-124 EVs. The EV concentration was 1×10^{12} particles mL^{-1}. GBM cells were immunostained for F-actin (Phalloidin, green). Migrated cell number, average migration distance, and invasion area of GBM cells toward the gel in a microfluidic device were investigated in triplicate. (*N* indicates total number of ROI, N indicates number of chips, * $p < 0.05$, ** $p < 0.01$,

*** $p < 0.001$). Bar charts are expressed as the mean ± S.D. Box-and-whisker plots show all individual points on the box.

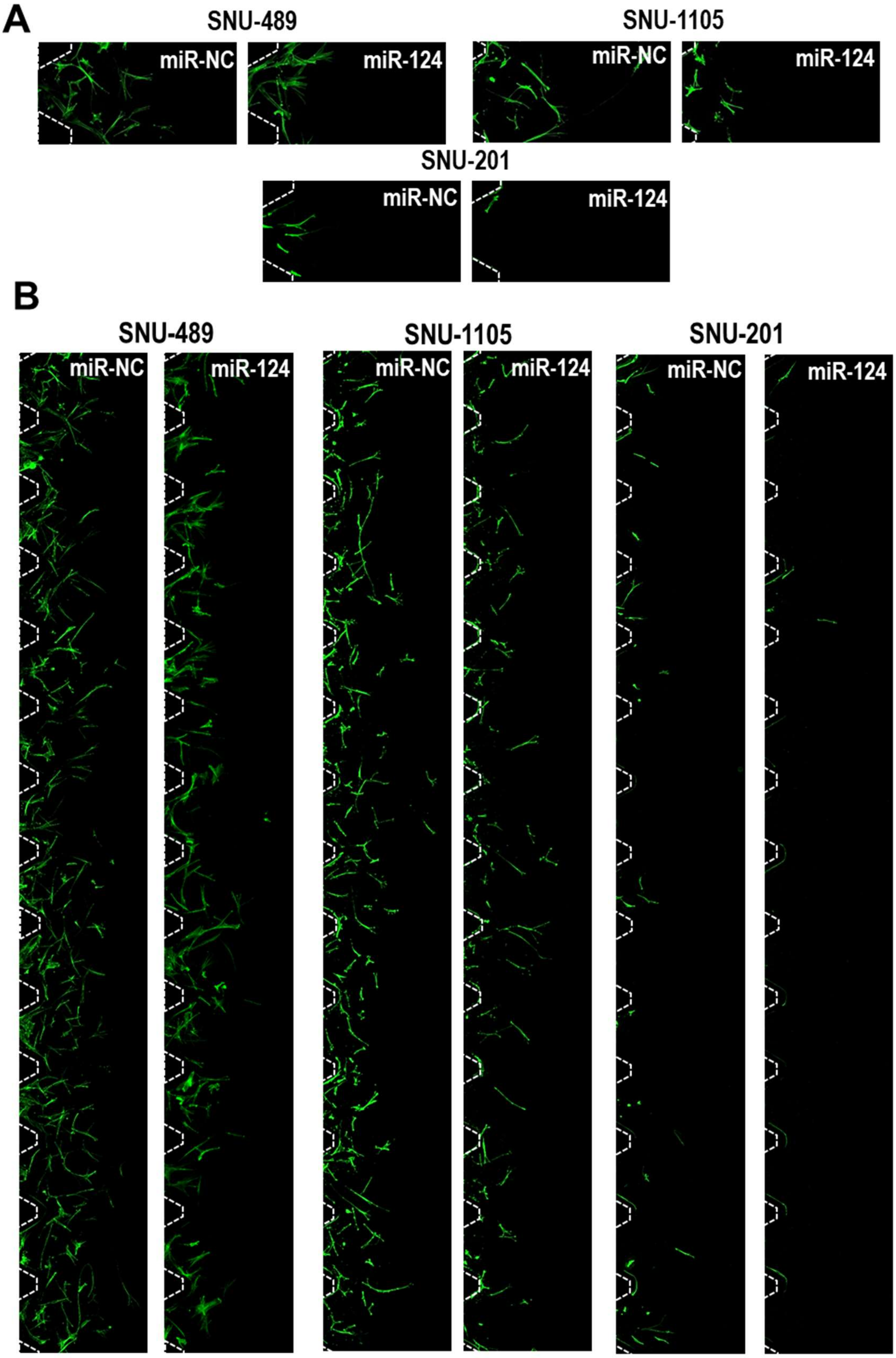
A
SNU-489
miR-NC
miR-124
SNU-1105
miR-NC
miR-124
SNU-201
miR-NC
miR-124
B
SNU-489
miR-NC
miR-124
SNU-1105
miR-NC
miR-124
SNU-201
miR-NC
miR-124

Figure S11. Images of patient-derived GBM cell lines treated with miR-124 EVs in a 3D microfluidic device. Images were compared to miR-NC-treated cells in a 3D microfluidic device. (A) Representative ROI images of GBM cell lines that were immunostained against F-actin. The experimental condition was the same as Figure S10. (B) Fully stitched image showing entire fields of ROIs in the microfluidic device. The 3D maximum projection images with Z-stack were stitched together.

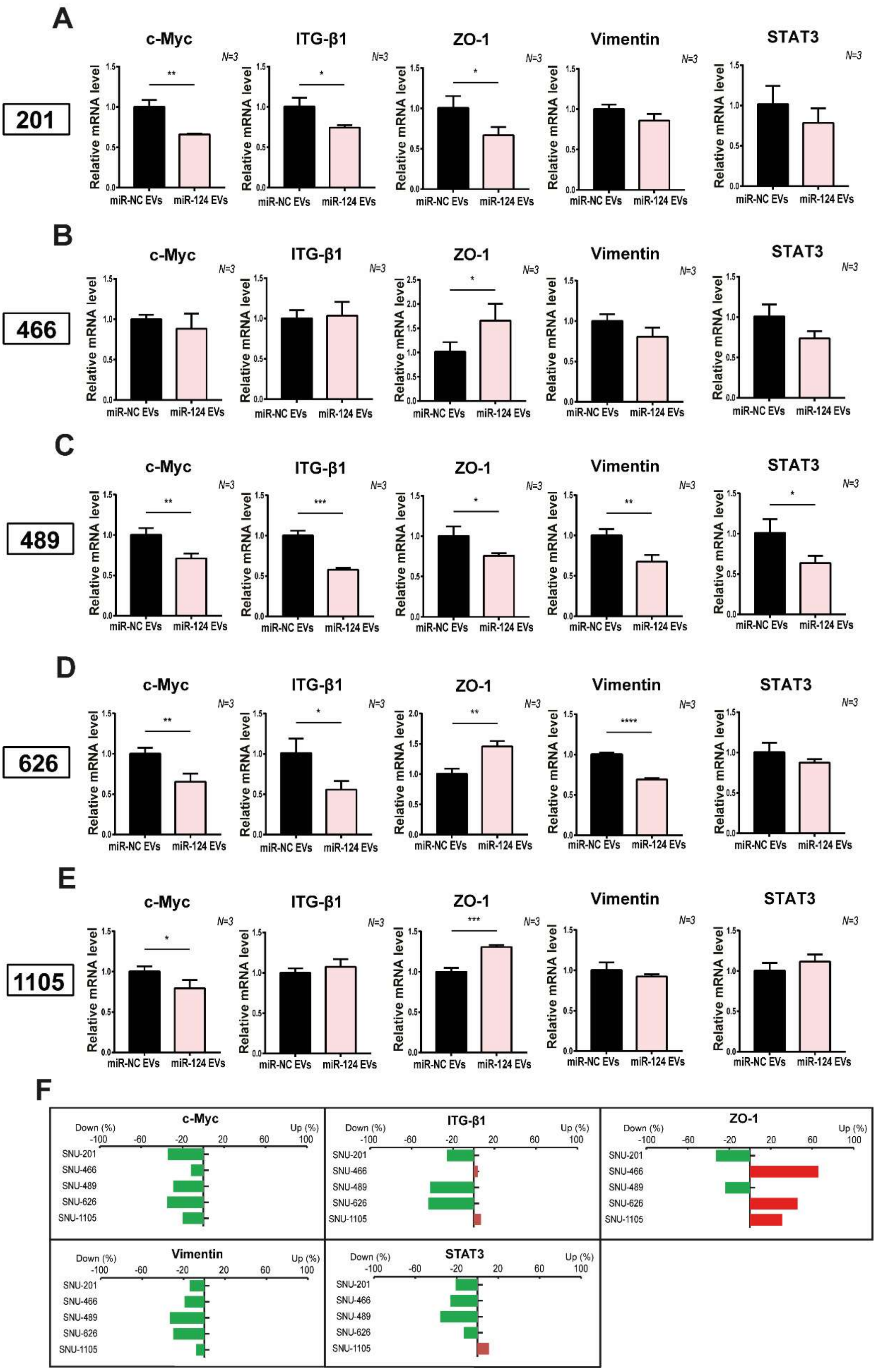
A
B
C
D
E
F
201
466
489
626
1105
c-Myc
ITG-β1
ZO-1
Vimentin
STAT3
N=3
Relative mRNA level
miR-NC EVs
miR-124 EVs
Down (%)
Up (%)
SNU-201
SNU-466
SNU-489
SNU-626
SNU-1105

Figure S12. Anti-tumor effect of miR-124 EVs in patient-derived GBM cell lines. Patient-derived GBM cell lines, (A) SNU-201, (B) SNU-466, (C) SNU-489, (D) SNU-626, and (E) SNU-1105 were cultured on a 96 well plate (7×10^4 cells mL^{-1}) and treated with miR-NC EVs or miR-124 EVs (1×10^{12} particles mL^{-1}) for 48 h. Relative mRNA expression levels of the proliferation marker *c-Myc*, migration-associated marker *ITG-β1*, epithelial marker *ZO-1*, mesenchymal marker *Vimentin*, and *STAT3* were investigated. GAPDH was used as an internal control. All values are expressed as the mean ± S.D. (* $p < 0.05$, ** $p < 0.01$, *** $p < 0.001$). (F) Comparison of gene expression by anti-tumor effects of miR-124 EVs in patient-derived GBM cell line. Up- and down-regulation of genes were indicated by red and green bars, respectively. The bar plots show the mean value of the triplicate data compared to the control.

References

1. Ghandi M, Huang FW, Jané-Valbuena J, Kryukov GV, Lo CC, McDonald ER, et al. Next-generation characterization of the cancer cell line encyclopedia. Nature. 2019; 569: 503-8.

2. Shin K-H, Choe G, Park Y-J, Jang J-H, Jung H-W, Park J-G. Establishment and characterization of nine human brain tumor cell lines. In Vitro Cell Dev Biol Anim. 2001; 37: 625-8.
